# Elevated N‐methyltransferase expression induced by hepatic stellate cells contributes to the metastasis of hepatocellular carcinoma via regulation of the CD44v3 isoform

**DOI:** 10.1002/1878-0261.12544

**Published:** 2019-07-11

**Authors:** Jie Li, Song You, Sheng Zhang, Qing Hu, Fuqiang Wang, Xiaoqin Chi, Wenxiu Zhao, Chengrong Xie, Changmao Zhang, Yaqi Yu, Jianmin Liu, Yue Zhao, Pingguo Liu, Yi Zhang, Xujin Wei, Qiu Li, Xiaomin Wang, Zhenyu Yin

**Affiliations:** ^1^ Department of Hepatobiliary Surgery ZhongShan Hospital of Xiamen University Fujian China; ^2^ Fujian Provincial Key Laboratory of Chronic Liver Disease and Hepatocellular Carcinoma ZhongShan Hospital of Xiamen University Fujian China; ^3^ Graduate College of Fujian Medical University Fuzhou Fujian China; ^4^ Department of Pathology, Hubei Cancer Hospital, Tongji Medical College Huazhong University of Science and Technology Wuhan Hubei China; ^5^ Medicine Clinical Laboratory Xiamen Xianyue Hospital Fujian China

**Keywords:** CD44, hepatic stellate cells, hepatocellular carcinoma, metastasis, NNMT

## Abstract

The cross‐talk between hepatic stellate cells (HSCs) and hepatic carcinoma cells contributes to hepatocellular carcinoma (HCC) progression, but the underlying mechanism is largely unknown. We report here that activated HSCs induce upregulation of nicotinamide N‐methyltransferase (NNMT), which is known to regulate multiple metabolic pathways in hepatoma cells of the liver. High levels of NNMT in HCC tissues were positively correlated with vascular invasion, increased serum HBV‐DNA levels, and distant metastasis. In addition, functional assays showed that NNMT promoted HCC cell invasion and metastasis by altering the histone H3 methylation on 27 methylation pattern and transcriptionally activating cluster of differentiation 44 (CD44). NNMT‐mediated N6‐methyladenosine modification of CD44 mRNA resulted in the formation of a CD44v3 splice variant, while its product 1‐methyl‐nicotinamide stabilized CD44 protein by preventing ubiquitin‐mediated degradation. Finally, NNMT was also shown to be a target of statins that inhibited metastasis of hepatoma cells. Taken together, our study shows for the first time that the NNMT/CD44v3 axis regulates HCC metastasis and presents NNMT as a promising prognostic biomarker and therapeutic target for HCC.

**Abbreviations**
1‐MNA: 1‐methyl‐nicotinamide
CD44: cluster of differentiation 44
CHX: cycloheximide
CM: conditioned medium
Co‐IP: coimmunoprecipitation
DEG: differentially expressed genes
DMEM: Dulbecco’s modified Eagle’s medium
GNMT: glycine N‐methyltransferase
H3K27: H3 methylation on 27
H3K9: H3 methylation on lysine 9
HCC: hepatocellular carcinoma
HSC: hepatic stellate cell
KD: knockdown
m6A: N6‐methyladenosine
NAM: nicotinamide
NNMT: nicotinamide N‐methyltransferase
OS: overall survival
RIP: RNA immunoprecipitation
RNA‐Seq: RNA sequencing
SAH: S‐adenosylhomocysteine
SAM: S‐adenosylmethionine

## 1 Introduction

Hepatocellular carcinoma (HCC) is the sixth most commonly diagnosed cancer and the third leading cause of cancer‐associated mortality worldwide (Chen *et al.*, 2016). The poor prognosis of HCC patients is largely attributed to the late diagnosis, and the high rates of metastasis, invasiveness, and recurrence of this cancer (Medavaram and Zhang, 2018). Therefore, it is vital to elucidate the mechanisms involved in HCC initiation and early metastasis and identify the early biomarkers and therapeutic targets.

More than 80% of liver cancers progress from inflammation‐related fibrosis/cirrhosis, which creates a preneoplastic microenvironment (Coulouarn and Clement, 2014). Hepatic stellate cells (HSCs), the stromal cells residing in the perisinusoidal space in the liver parenchyma, are the major cells involved in the progression of chronic hepatitis, liver fibrosis, and cirrhosis to liver cancer. The interaction between HSCs and HCC cells contributes to tumor growth, metastasis, angiogenesis, and immunosuppression by cytokines. (Thompson *et al.*, 2015; Xu *et al.*, 2016). However, the exact mechanism of HSC‐mediated progression of HCC, especially invasion and metastasis, is still unclear.

Nicotinamide N‐methyltransferase (NNMT) was originally identified as the enzyme responsible for the methylation of Nicotinamide (NAM), a form of vitamin B3 (Aksoy *et al.*, 1994; Cantoni, 1951). In this study, human hepatoma cell lines showed a significant upregulation in NNMT mRNA levels when treated with HSC‐derived conditioned media (CM). Recent studies show that NNMT regulates post‐translational protein acetylation and (histone) methylation by, respectively, modulating Sirt1 deacetylase activity and the levels of the methyl donor S‐adenosylmethionine (SAM) (Ulanovskaya *et al.*, 2013). In contrast, other studies showed that NNMT had no effect on the SAM/S‐adenosylhomocysteine (SAH) ratio in hepatocytes due to the high levels of glycine N‐methyltransferase (GNMT) in these cells (Hong *et al.*, 2015; Mudd *et al.*, 2007). In addition, NNMT is also highly expressed in several tumor types, indicating an oncogenic function. Studies on lung cancer (Bach *et al.*, 2018), bladder cancer (Wu *et al.*, 2008), gastric cancer (Lim *et al.*, 2006), and pancreatic cancer (Yu *et al.*, 2015) have shown that NNMT is closely related to the tumor stage, metastasis, and chemo‐resistance. However, an inverse correlation between NNMT expression levels and tumor size has been reported in pancreatic cancer and oral squamous cell carcinoma (Emanuelli *et al.*, 2010). NNMT is most abundant in the liver, where it regulates multiple metabolic pathways (Hong *et al.*, 2015), and is significantly downregulated in HCC tissues relative to normal adjacent tissues. However, within the HCC samples, higher levels of NNMT were positively associated with poor prognosis (Aksoy *et al.*, 1994; Kim *et al.*, 2009). Recent evidence indicates that NNMT is upregulated by TGF‐β in gastric and renal cancer cells and promotes their metastasis (Campagna *et al.*, 2018; Liang *et al.*, 2018). Nevertheless, the role of NNMT in liver cancer cell invasion and metastasis has not been elucidated so far.

The transmembrane glycoprotein receptor cluster of differentiation 44 (CD44) (Bourguignon *et al.*, 2017; Krolikoski *et al.*, 2018) binds to various cell surface ligands and enables cell‐to‐cell and cell‐to‐matrix adhesion, which is essential for tumor cell metastasis and progression. Indeed, studies show a strong correlation between high CD44 expression levels and tumor metastasis, invasion, and prognosis (Bourguignon *et al.*, 2017). CD44 has multiple splice variants, including the ubiquitous V4, which along with V3 and V6 is closely related to tumor metastasis (Gutjahr *et al.*, 2018; Matsumoto and Itou, 2018). N6‐methyladenosine (m6A) is a common mRNA modification that occurs at the consensus RRACH motif (R = G or A; H = A, C, or U) and is enriched near the stop codons (Dominissini *et al.*, 2012). It is a reversible modification controlled by the balance between RNA methyltransferase and demethylases, with the former comprising of the METTL3, METTL14, WTAP (Bokar *et al.*, 1997; Fu and Ares, 2014), FTO (Jia *et al.*, 2011), and ALKBH5 (Zheng *et al.*, 2013) subunits. The m6A modification regulates various biological processes like stem cell behavior, miRNA origin, RNA–protein interaction, and mRNA expression, translation, stability, and alternative splicing (Gilbert *et al.*, 2016; Jiang *et al.*, 2017). CD44 is also a stem cell marker that is upregulated by NNMT (Jung *et al.*, 2017), although the mechanistic basis is unknown. We hypothesized that aberrant methylation in the HCC cells mediated by dysregulated NNMT may alter m6A abundance in the CD44 mRNA, thereby increasing the proportion of CD44V3 splice variant in these cells and accelerating tumor progression.

We found that activated HSCs increased NNMT levels in the HCC cells, which was positively correlated with vascular invasion, high serum HBV levels, and distant metastasis. Mechanistically, the aberrant activity of NNMT altered methylation patterns of histone H3 methylation on 27 (H3K27) and CD44 mRNAs, resulting in higher levels of the CD44V3 isoform. In addition, its product 1‐methyl‐nicotinamide (1‐MNA) stabilized CD44 protein by preventing ubiquitin‐mediated degradation. NNMT was also a target of statins, which inhibited metastasis of hepatoma cells. Therefore, NNMT is a promising prognostic marker and therapeutic target for HCC.

## 2 Materials and methods

### 2.1 Cell lines and cell culture

The human HCC cell lines were obtained from the cell bank of Shanghai Institute of Cell Biology and cultured in Dulbecco’s modified Eagle’s medium (DMEM) (Hy Clone) supplemented with 10% FBS (Gibco, Grand Island, NY, USA), 100 IU·mL^−1^ penicillin, and 100 μg·mL^−1^ streptomycin (Millipore, Billerica, MA, USA). In general, the researchers believe that SMMC‐7721, MHCC‐97H, LM3, and QGY‐7701 cell lines have higher invasive ability than ATCC‐HepG2, PLC/PRF/5, and Huh7 cell lines. The SMMC‐7721, QGY‐7701, Huh7, BEL‐7402 cell lines proliferation ability are stronger than other liver cancer cell lines. The HSC cell line Lx2 was purchased from Merck Millipore and cultured in DMEM supplemented with 2% FBS (Gibco, Certified, US Origin) and the antibiotics. Quiescent HSCs were activated *in vitro* by incubating the cells with 10 ng·mL^−1^ TGF‐β for 48 h and partly restored to quiescence by 10 ng·mL^−1^ calcipotriol. The FBS‐free HSC‐CM was collected, centrifuged, and stored at −80 °C. HCC cells and HSC cells were cocultured through a coculture chamber, in which HSC cells(2 × 10^5^ cells) were cultured in the upper layer and HCC cells(2 × 10^5^ cells) were cultured in the lower layer. In addition, the intermediate membrane allows the passage of culture fluid rather than cells. The subsequent experiment was to collect HSCs culture supernatant CM and HCC medium and mix with 1 (5 mL): 1 (5 mL) to simulate HCC cells in the coculture environment.

### 2.2 Real‐time PCR

Real‐time PCR was performed using the qPCR Master Mix (Roche, Shanghai, China) and LightCycker^®^96 SW 1.1 (Roche) according to the manufacturer´s instructions. The gene‐specific primer sequences are shown in Table S1.

### 2.3 Western blotting

Protein samples were extracted from tissues and cells using RIPA lysis buffer (Beyotime, Shanghai, China) supplemented with a protease inhibitors cocktail. The protein lysates were separated by SDS/PAGE and transferred to PVDF membranes that were blocked with skim milk powder at room temperature for an hour. The blots were then incubated overnight with primary antibodies targeting NNMT (1 : 1000, 15123‐1‐AP; Proteintech, Wuhan, China), CD44 (1 : 1000, #3570; CST, Danvers, MA, USA), GNMT (1 : 1000,18790‐1AP; Proteintech), H3K27me3 (1 : 1000, A2363; Abclonal, Wuhan, China), β‐actin (1 : 1000, AT0001; CMCTAG), FTO (1 : 1000, ab92821; Abcam, Cambridge, MA, USA), and ALKBH5 (1 : 1000,16837‐1‐AP; Proteintech). Following incubation with the secondary antibody at room temperature for an hour, the bands were visualized using Immobilon TM Western (Millipore).

### 2.4 Luciferase reporter assay

The CD44 reporter vector and the Renilla luciferase plasmid were cotransfected into the HEK293T cells at a ratio 10 to 1, along with NNMT, scrambled shRNA, and shNNMT‐expressing or empty plasmids. After 24 h, the cells were harvested, lysed, and analyzed with the Dual‐luciferase reporter assay kit (Promega, Madison, WI, USA) according to the manufacturer’s instructions. The average ratio of firefly luciferase and Renilla luciferase activities was calculated from triplicate tests of three independent experiments.

### 2.5 Immunofluorescence analysis

Cells were seeded onto 12‐mm cover slips and fixed with 4% paraformaldehyde (Beyotime) for 20 min at 4 °C, permeabilized with 0.25% Triton X‐100 (Millipore) at room temperature for 30 min, and then blocked with 2% BSA (Gibco) at room temperature for 60 min. The fixed cells were incubated overnight with DAPI (1 : 1000) and Phalloidin (1 : 750) at 4 °C. After washing thrice with TBST buffer, the stained cells were observed by confocal immunofluorescence microscopy (Zeiss, Jena, Germany).

### 2.6 Migration and invasion analysis

Cell migration and invasion were analyzed using the 24‐well polycarbonate membrane cell migration assay kit (#3422; BD Biosciences, San Jose, CA, USA) according to the manufacturer’s instructions. Briefly, 2 × 10^5^ HCC cells were seeded in the upper chambers of the membrane insert with serum‐free medium, and the lower chambers were each filled with 800 μL medium supplemented with 15% FBS. After 48 h, the migrated cells on the lower surface of the membrane were fixed and stained with crystal violet, and counted in five random microscopic fields per well with the double‐blind method. Cell invasion was assayed using BD BioCoat™ Matrigel™ Invasion Chambers (#354480; BD Biosciences) following the same protocol as above, except that 5 × 10^5^ HCC cells were seeded and the upper chambers were precoated with ECMatrix™ gel.

### 2.7 Cell viability e analysis

Cell viability was evaluated with the Counting Kit‐8 (CCK‐8) Kit (#YB‐K001; Yi Yuan Biotechnologies, Guangzhou, China) according to the manufacturer’s instructions. The optical density (OD) at 450 nm was measured using a microplate reader, and the average of three independent experiments was calculated.

### 2.8 Coimmunoprecipitation

Equal amounts of cell lysates were incubated overnight with control IgG or specific primary antibodies and 40 μL protein A/G‐agarose at 4 °C. After washing five times (15 min/time) with IP lysis buffer, the immuno‐precipitated complex was centrifuged and eluted from the beads by boiling in 1 × SDS loading buffer. Subsequently, the precipitates were probed by western blotting. To assess ubiquitylation, the protein lysates were incubated with the antiubiquitination antibody and then probed with antibodies targeting β‐actin, CD44, and NNMT.

### 2.9 RNA immunoprecipitation (RIP)

The m6A RNAs were immuno‐precipitated using Sera‐Mag Oligo (dT)‐Coated Magnetic particles as per the manufacturer’s instructions. Briefly, 150 mg of RNA samples was treated with RNase H, precipitated, and resuspended in 20 mL water, 130 mL IP buffer (10 mm Tris pH 7.5, 150 mm NaCl, 0.1% Igepal), 0.5 mL RNase in Plus, and 1mg of IgG or anti‐m6A antibody. After nutating the mixture for 1 h at 4 °C, 15 mL of washed Protein A Dyna beads was added to each sample and nutated for 1 h. The beads were washed five times with IP buffer, and the bound RNA was eluted with 200 mL G‐50 buffer supplemented with 0.1 mg·mL^−1^ Proteinase K and incubated at 37 °C for 1 h. The RNA–protein complexes were extracted with PCA and ethanol precipitated, and then analyzed by RT/PCR.

### 2.10 Ch‐IP

Ch‐IP was performed using the Magna Chip TM Hisens Kit (Millipore) according to the manufacturer´s instructions. Briefly, the DNA–protein complexes were sonicated into ~ 500‐bp DNA fragments, and the CD44 sequences cross‐linked to H3K27me3 were detected using real‐time PCR. The sequences of CD44 primers are listed in Table S1.

### 2.11 Patients and clinical specimens

The Chronic Liver Disease Biological Sample Bank, Department of Hepatobiliary Surgery, ZhongShan Hospital, Xiamen University, provided 92 paired HCC and matched adjacent normal tissue samples and follow‐up patient information for this study. Written informed consent was obtained from each patient, and the study protocol followed the Ethical Guidelines of the Declaration of Helsinki and was approved by the Ethics Committee of the institute.

### 2.12 Hematoxylin–eosin (HE) staining and immunohistochemistry (IHC)

Hematoxylin–eosin staining and IHC were performed as described previously (Zhang *et al.*, 2015), with some modifications according to the antibody instructions. Antibodies targeting NNMT (1 : 2000, 15123‐1‐AP; Proteintech) and CD44V3 (1 : 800, ab34229; Abcam) were used, and the *in situ* protein expression levels were quantified in terms of integrated OD values.

### 2.13 RNA‐Seq and mass spectrometry

RNA sequencing (RNA‐Seq) and mass spectrometry services were provided by Kiddio Biotech and Xiamen University of Physics and Mechanical Engineering.

### 2.14 RNA stability assay

Hepatocellular carcinoma cells treated with 5 mg·mL^−1^ actinomycin D (Meilun Biology, Dalian,China) were harvested at the different time points and subjected to real‐time PCR.

### 2.15 Animal studies

Male 5‐week‐old specific pathogen‐free nude mice were provided by the Laboratory Animal Center of Xiamen University. All procedures were conducted in accordance with the regulations and internal biosafety and bioethics guidelines of Xiamen University. SMMC‐7721 or MHCC‐97H cells (1 × 10^6^) in 25 µL serum‐free medium containing 5 µL Matrigel were inoculated to the liver and subcutaneous region of the mice, respectively. After 8 weeks, the mice were sacrificed and liver/pulmonary metastatic foci were observed by HE staining.

### 2.16 Statistical analysis

Data were presented as the mean ± SD and analyzed using the ibm spss 21.0 software (IBM, Armonk, NY, USA). Significant differences between groups were determined using *t*‐test (two‐tailed unpaired) when the data met the criteria of normal distribution as per D’Agostino test. Patient survival was determined by the Kaplan–Meier method. Chi‐square test was used for correlation analysis, and two‐way ANOVA for multiple comparisons. *P* < 0.05 was considered statistically significant.

## 3 Results

### 3.1 Activated HSCs increase HCC invasion and migration by upregulating NNMT

**Figure 1:**
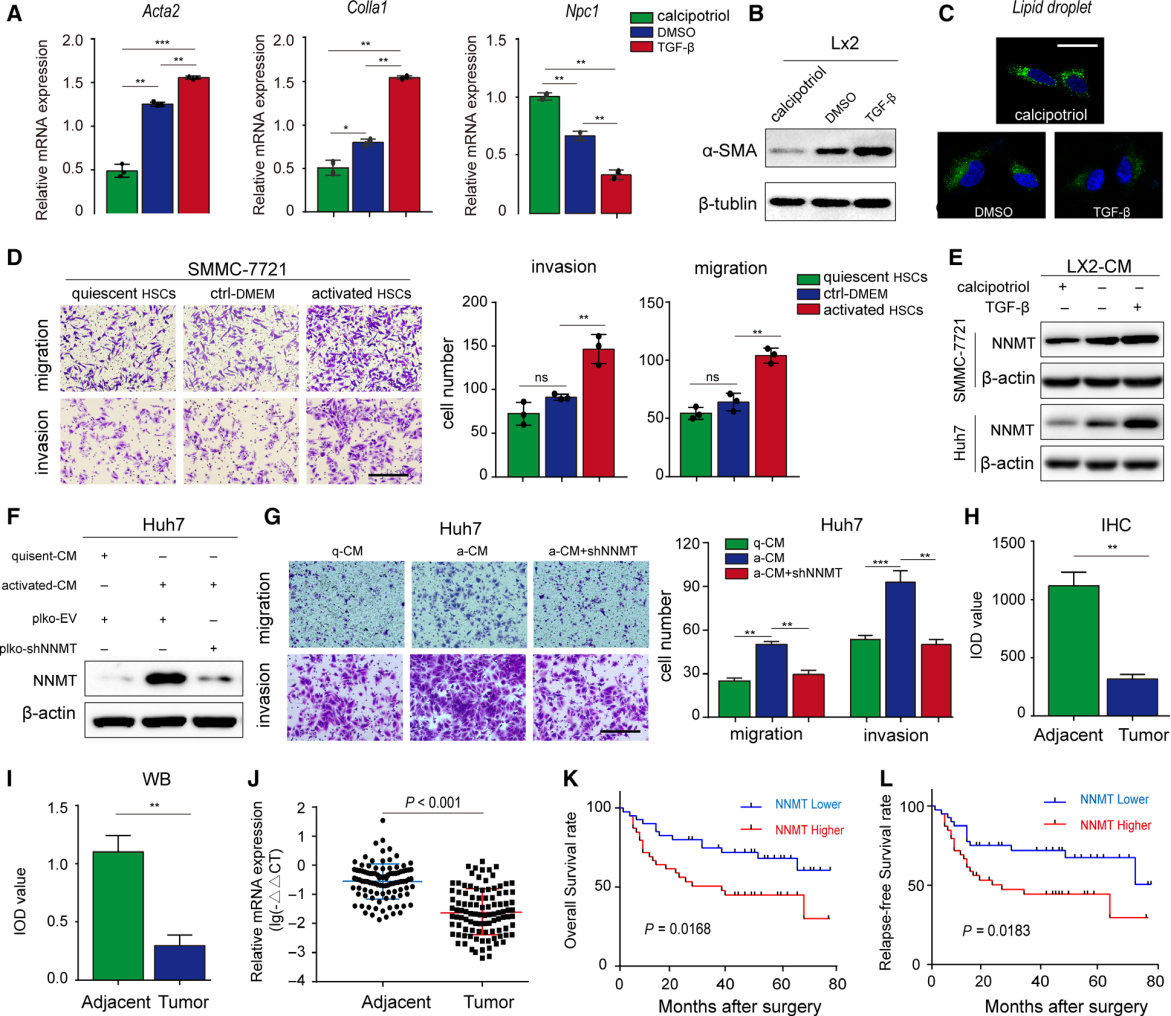
Activated HSCs increase HCC migration and invasion by upregulating NNMT. (A–B) TGF‐β upregulated ACTA2, Colla1, and a‐SMA and downregulated NPC1 in the LX2 cells. (C) Number of BODIPY493/503‐stained lipid droplets decreased in LX2 cells upon activation. (D) The *in vitro* migration and invasion of SMMC‐7721 cells cocultured with HSCs were assessed by the Transwell assay. (E) Western blot images showing expression of NNMT in SMMC‐7721 and Huh7 cell lines cocultured with HSCs. (F–G) Efficiency of NNMT KD in Huh7 cells cocultured with HSCs and its effect on migration and invasion. (H) In situ NNMT protein expression in 92 HCC and corresponding adjacent tissues, and the IOD values (original magnification ×200). (I) Immunoblot showing NNMT protein expression in paired HCC tumor and adjacent samples and the IOD values. (J) Relative expression of NNMT mRNA in the paired HCC tumor and adjacent tissues. (K‐L) Kaplan–Meier curves showing OS and relapse‐free survival in NNMT‐high (*n* = 46) and NNMT‐low (*n* = 46) patients. The *t*‐test was used in A, D, G. The two‐related samples Wilcoxon nonparametric test was used in H, I, J. The error bars represent SD. Scale bars = 200 μm. Each experiment was performed in triplicates. ***P < 0.001, **P < 0.01, and *P < 0.05.

Quiescent HSCs were activated *in vitro* with TGF‐beta (10 ng·mL^−1^, 48 h) and partly restored to quiescence by calcipotriol (10 ng·mL^−1^) (Duran *et al.*, 2016). HSC activation was indicated by the upregulation of a‐SMA, *acta2,* and *col1a1*, downregulation of *npc2*, and a gradual reduction in intracellular lipid droplets (Fig. 1A–C). In our previous study, we found that activated HSCs promoted HCC development (Xu *et al.*, 2016). To further elucidate the mechanism, we cultured the SMMC‐7721 cells with the CM of activated and quiescent HSCs and found that the former significantly promoted HCC cell migration and invasion (Fig. 1D). In addition, RNA‐Seq and western blotting analysis showed that activated rather than quiescent HSC‐CM significantly upregulated NNMT mRNA levels in the HCC cell lines (Figs S2A and 1E). Consistent with the findings so far, knockdown (KD) of NNMT in Huh7 cells attenuated the activated HSC‐CM‐induced migration and invasion (Fig. 1F–G). Taken together, activated HSCs enhance HCC invasion and migration by upregulating NNMT. Based on our and others’ findings, we hypothesized that activated HSCs exerted these effects by secreting TGF‐β.

### 3.2 NNMT expression is positively correlated with poor prognosis of HCC

We examined NNMT levels in 92 pairs of HCC and adjacent tissue samples, and detected significant downregulation in NNMT protein (*P* < 0.01; Figs 1H‐I and S1A‐C) and mRNA (Fig. 1J) levels in HCC tissues compared to the nontumor tissues. Based on the IOD values of IHC staining, the patients were divided into the NNMT‐high (*n* = 46) and NNMT‐low (*n* = 46) groups (Fig. S1B), and the former had significantly shorter overall survival (OS) (Fig. 1K, *P* = 0.0168) and tumor‐free survival (Fig. 1L, *P* = 0.0183) rates. Therefore, although NNMT was downregulated in HCC tissues relative to normal adjacent tissues, the expression levels of NNMT in the HCC tissues were positively associated with poor prognosis. We analyzed the correlation between NNMT expression and clinicopathological factors using the chi‐square test and found that higher NNMT expression was significantly correlated with unfavorable prognostic features such as vascular invasion (*P* = 0.006), liver cirrhosis status (*P* = 0.011), serum HBV levels (*P* = 0.029), and distant metastasis (*P* = 0.001) (Table 1). Taken together, our results indicate NNMT may accelerate HCC progression by promoting invasion and metastasis.

**Table 1:** NNMT higher expression correlates with clinic–pathological factors of HCC patients. *P* < 0.05 was regarded as statistically significant.

Clinical–pathology factors	NNMT higher expression	*N*	X^2^	*P*
Age(year)				
＜ 50	18 (47.4%)	38	0.179	0.416
≥ 50	28 (51.9%)	54		
Gender				
Male	34 (45.9%)	74	2.486	0.094
Female	12 (66.7%)	18		
Tumor size				
＜ 5cm	32 (47.8%)	67	0.494	0.320
≥ 5cm	14 (56.0%)	25		
Metastasis				
No	29 (40.8%)	71	10.428	0.001**
Yes	17 (80.9%)	21		
Microinvasion				
No	18 (36.7%)	49	7.379	0.006**
Yes	28 (65.1%)	43		
Differentiation				
Poor	7 (53.8%)	13	0.331	0.188
Moderate	36 (48.6%)	74		
Well	3 (60.0%)	5		
Liver cirrhosis status				
No	17 (36.9%)	46	6.261	0.011*
Yes	29 (63.0%)	46		
Serum HBV level (cps·mL^−1^)				
＜ 1000	13 (48.1%)	27	4.639	0.029*
≥ 1000	33 (73.3%)	45		
Serum AFP level (µg·L^−1^)				
＜ 400	24 (46.2%)	52	0.708	0.264
≥ 400	22 (55.0%)	40		

** *P* < 0.01.

* *P* ^< 0.05.^

### 3.3 Activated HSCs promote invasion and metastasis of HCC cells by upregulating CD44 via NNMT

**Figure 2:**
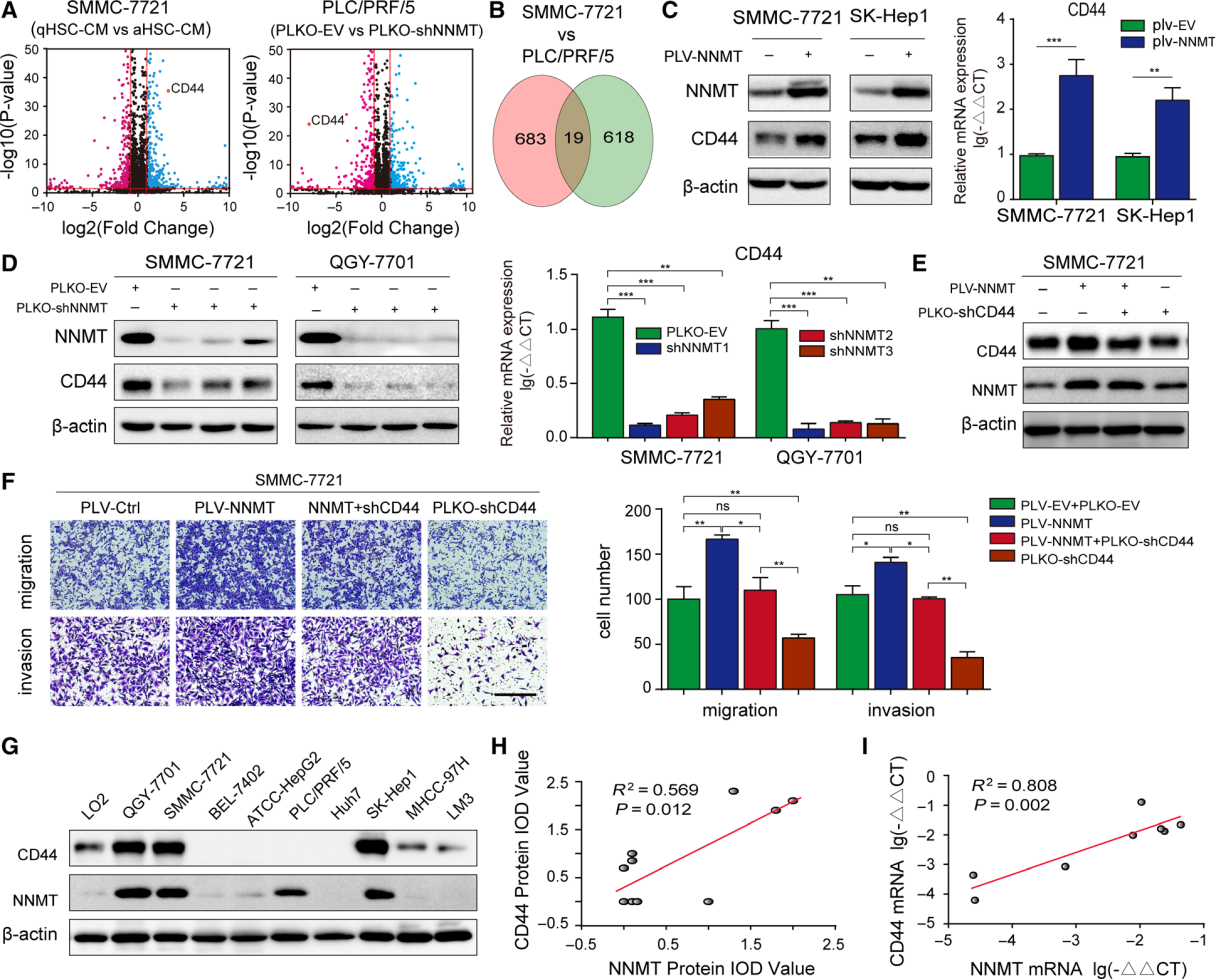
Activated HSCs upregulate CD44 via NNMT to promote invasion and metastasis. (A) Volcano map analysis of the DEGs in SMMC‐7721 cells treated with activated HSCs‐CM, quiescent HSCs‐CM, and in the plko‐ and NNMT‐KD PLC/PRF/5 cells. (B) Wayne diagram showing the DEGs common in both cell lines. (C) NNMT and CD44 expression levels in SMMC‐7721 and SK‐Hep1 cell lines transduced with the NNMT‐overexpressing lentivirus. (D) Efficiency of NNMT KD and CD44 expression in NNMT‐KD SMMC‐7721 and QGY‐7701 cells. (E) CD44 expression levels in the NNMT‐overexpressing and CD44‐KD SMMC‐7721 cells. (F) Transwell assay showing the invasion and metastatic ability of control, NNMT, NNMT + CD44‐KD, and CD44‐KD cells. (G) Relative expression of NNMT and CD44 proteins in L02 hepatocytes and HCC cell lines. (H) Regression analysis of CD44 and NNMT protein expression in the cell lines. (I) Regression analysis of CD44 and NNMT mRNA expression in hepatoma cell lines. The *t*‐test was used in C, D, F. The error bars represent SD. Scale bars = 200 μm. Each experiment was performed in triplicate. ***P < 0.001, **P < 0.01, and *P < 0.05.

To further explore the molecular basis of the interplay between activated HSCs and HCC cells, we knocked down NNMT in the PLC/PRF/5 cells lines and analyzed the expression levels of the downstream genes. We detected 618 differentially expressed genes (DEGs) in the NNMT‐KD PLC/PRF/5 cells and 683 DEGs in the SMMC‐7721 cells incubated with activated HSC‐CM. Nineteen DEGs were common to both (Figs 2A–B and S2A) and included CD44 which is regulated by NNMT in various tumors (Jung *et al.*, 2017; Shlomi and Rabinowitz, 2013). Ectopic expression of NNMT in SMMC‐7721 and SK‐Hep1 cells significantly upregulated CD44 (Fig. 2C), while its KD had the opposite effect (Fig. 2D). To determine whether NNMT enhanced HCC cell invasion via CD44, we knocked down CD44 in the NNMT‐overexpressing SMMC‐7721 cells and found that it significantly reversed the prometastatic effects of NNMT (Fig. 2E‐F). In addition, we examined the expression levels of NNMT and CD44 in a battery of HCC cell lines (Fig. 2G) and found a significant positive correlation between them (Fig. 2H). Interestingly, no significant correlation was seen between CD44 and NNMT mRNA levels when all cell lines were considered (Fig. S2B), but upon excluding LO2 and HepG2, a significant positive correlation was seen (Fig. 2I). Taken together, the NNMT induced by activated HSC‐CM facilitates HCC metastasis by upregulating CD44.

### 3.4 NNMT regulates intracellular methylation potential during GNMT downregulation or deficiency

**Figure 3:**
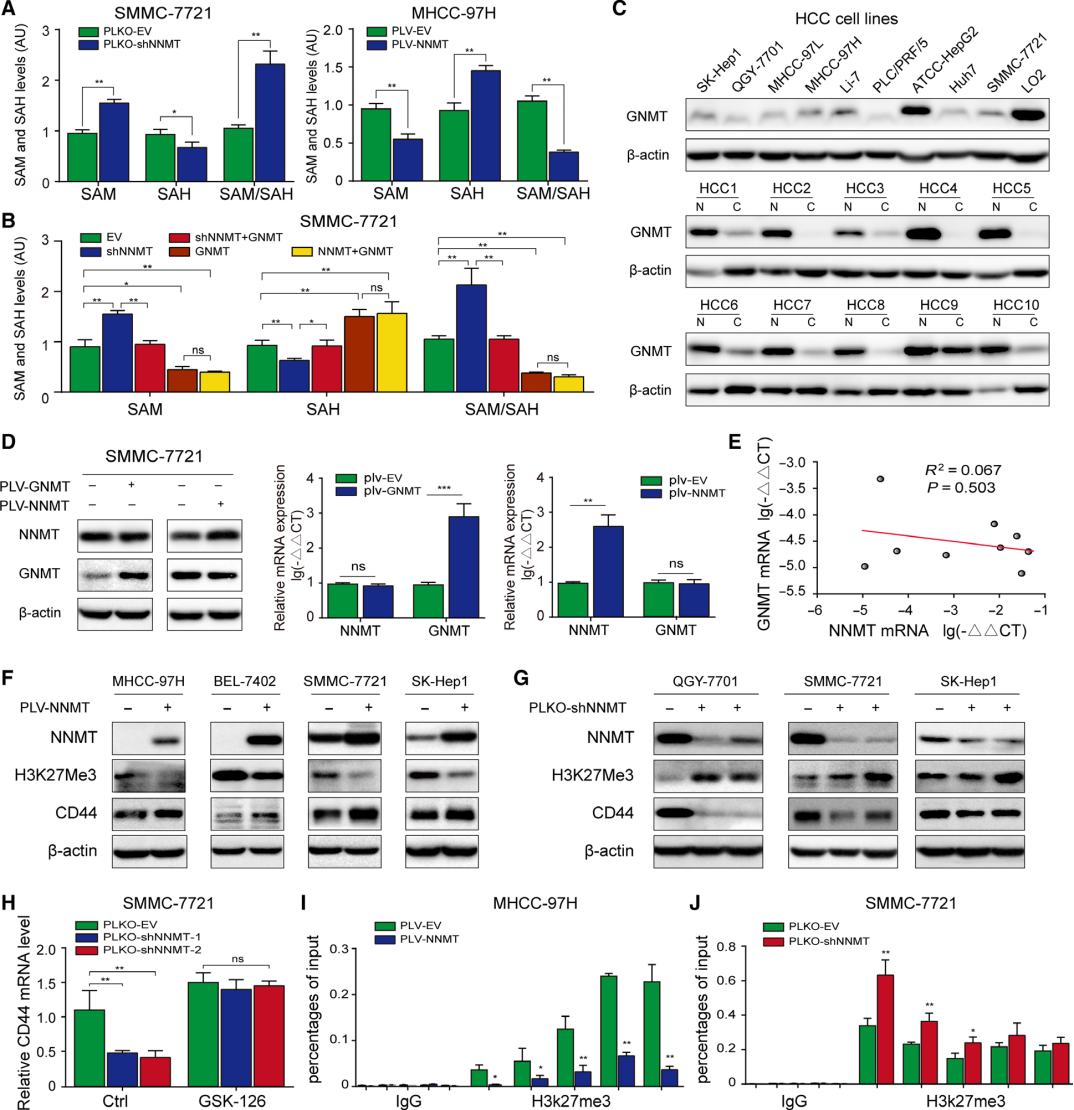
NNMT regulates intracellular methylation potential and CD44 transcription by inhibiting histone H3K27 methylation. (A) Mass spectrometric detection of relative SAM and SAH content and their ratio in NNMT‐KD SMMC‐7721 and NNMT‐MHCC‐97H cells. (B) Mass spectrometric detection of relative SAM and SAH content and their ratio in EV (empty vector), shNNMT, shNNMT + GNMT, GNMT and NNMT + GNMT cells. (C) GNMT protein expression in HCC cell lines, liver cancer tissues, and paracancerous tissues. (D) The effect of GNMT/NNMT overexpression on the expression levels of the other in 7721 cells. (E) Regression analysis of GNMT and NNMT mRNA expression in hepatoma cell lines. (F–G) H3K27me3 levels in the NNMT‐overexpressing and KD cells. (H) CD44 mRNA levels in control and NNMT‐KD SMMC‐7721 cells after treatment with GSK‐126 (5 mm for 72 h). (I–J) ChIP‐qPCR analyses of H3K27me3 binding on CD44 promoter in NNMT‐MHCC‐97H and NNMT‐KD SMMC‐7721 cells. The *t*‐test was used in A, B, D, H, I, G. The error bars represent SD. Each experiment was performed in triplicate. ***P < 0.001, **P < 0.01, and *P < 0.05

Nicotinamide N‐methyltransferase significantly modulates intracellular methylation potential (Shlomi and Rabinowitz, 2013), although its role in histone methylation is ambiguous. Several studies correlate the inability of NNMT to alter the SAM/SAH ratio in hepatocytes to the high levels of GNMT in these cells (Hong *et al.*, 2015; Mudd *et al.*, 2007). Therefore, we determined whether NNMT affected the methylation potential in HCC cells using mass spectrometry analysis. NNMT KD significantly slowed the conversion of SAM to SAH in the SMMC‐7721 cells resulting in an increase in SAM/SAH ratio. Conversely, overexpression of NNMT aided this conversion in MHCC‐97H cells and lowered the SAM/SAH ratio (Fig. 3A). In agreement with the previous studies, we did not detect these changes in the GNMT‐expressing normal liver LO2 and HepG2 cell lines (Fig. S3A‐B), which likely explains the lack of any correlation between the CD44 and NNMT mRNA levels in these cells (Fig. S2A). Furthermore, GNMT levels were lower in the HCC cells and tissues (Fig. 3C), and its ectopic expression restored the SAM/SAH ratio in NNMT‐KD SMMC‐7721 cells. However, it did not further decrease the ratio of SAM/SAH in NNMT‐overexpressing cells (Fig. 3B). Finally, NNMT and GNMT did not affect each other’s expression levels in SMMC‐7721 cells (Fig. 3D), and their mRNA levels were not correlated in hepatoma cell lines (Fig. 3E). These results suggest that NNMT regulates the intracellular methylation potential when GNMT is expressed at relatively low levels or is absent.

### 3.5 NNMT transcriptionally regulates CD44 by modifying histone H3K27 methylation

To further analyze NNMT‐mediated transcriptional regulation of CD44, we performed CD44 promoter‐driven luciferase reporter assays. Neither NNMT KD nor overexpression significantly affected luciferase reporter activity (Fig. S4A), indicating that NNMT does not directly regulate CD44 promoter activity. In addition, altered NNMT expression did not significantly affect the rate of CD44 mRNA degradation in the presence of the transcription inhibitor actinomycin D (Fig. S4B), indicating that NNMT also does not target CD44 mRNA stability. Given that NNMT regulates the intracellular methylation potential and CD44 is regulated by histone H3K27 (Wang *et al.*, 2016), we hypothesized that NNMT indirectly activates CD44 transcription by inhibiting H3K27 methylation. As shown in Fig. 3F, ectopic expression of NNMT significantly attenuated H3K27me3 levels and upregulated CD44 in MHCC‐97H, BEL‐7402, SMMC‐7721 and SK‐Hep1 cells, while NNMT KD had the opposite effects in the QGY‐7701, SMMC‐7721, and SK‐hep1 cells (Fig. 3G). Consistent with our hypothesis, the histone methylation inhibitor GSK‐126 restored CD44 mRNA levels in NNMT‐KD SMMC‐7721 cells (Fig. 3H). Furthermore, ChIP assay showed that NNMT overexpression and KD, respectively, diminished and increased H3K27me3 levels at the CD44 promoter (Fig. 3I‐J). However, there was no evidence of direct binding of NNMT to the CD44 promoter (Fig. S5A‐B), which explains the lack of response of the reporter construct to NNMT overexpression or KD (Fig. S4A). Taken together, NNMT promotes CD44 transcription by modulating histone H3K27 methylation.

### 3.6 NNMT‐mediated regulation of CD44 mRNA m6A promotes its splicing into the CD44v3 isoform

**Figure 4:**
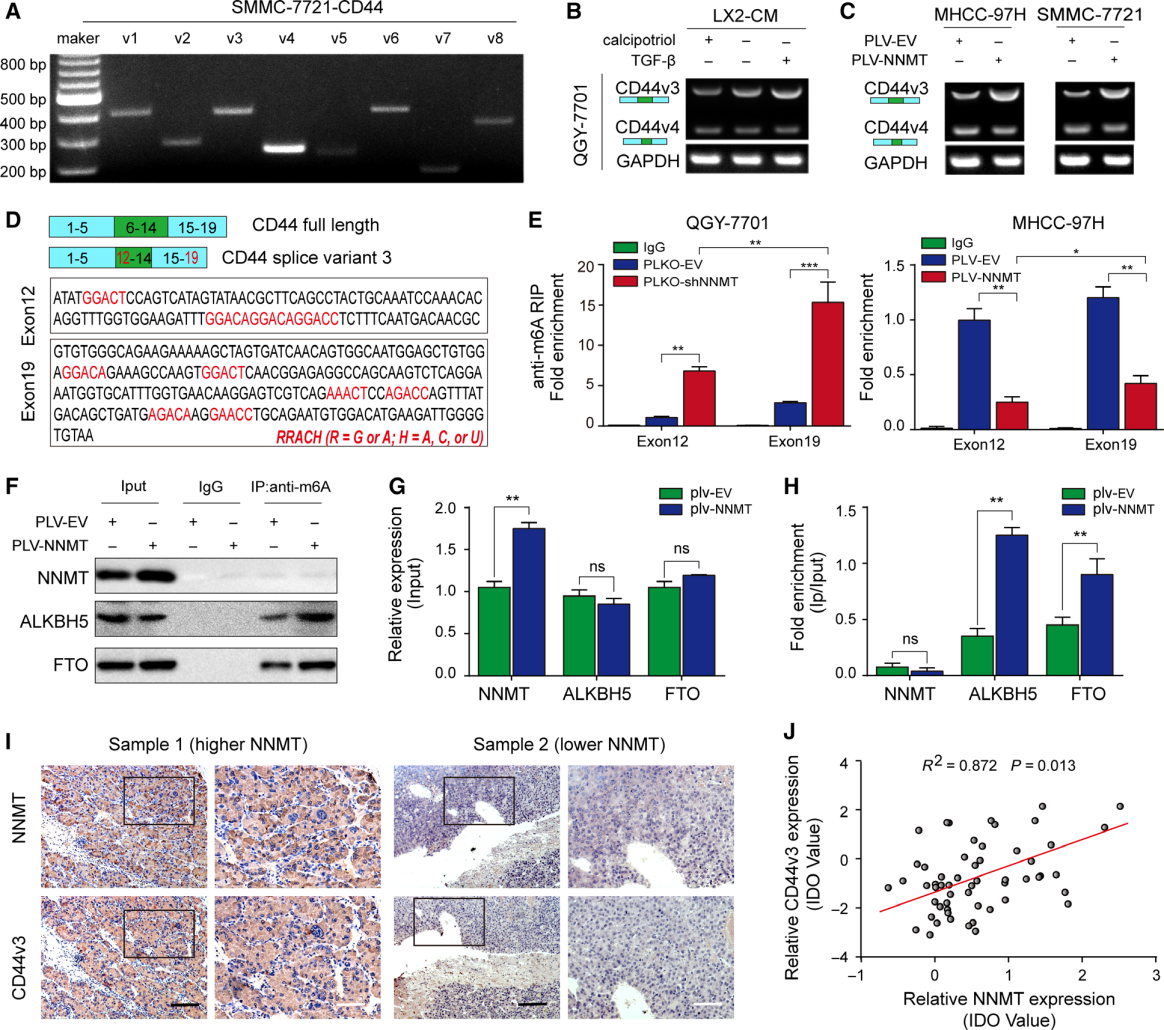
NNMT‐mediated CD44 mRNA m6A modification induces CD44v3. (A) Northern blot showing expression levels of CD44v1‐6 mRNA in SMMC‐7721 cells. (B). The expression levels of CD44v3 and v4 transcripts in QGY‐7701 cells treated with quiescent HSC‐CM, HSC‐CM, and activated HSC‐CM. (C) The CD44v3 and v4 expression levels in NNMT‐overexpressing MHCC‐97H and MMC‐7721 cells. (D) Analysis of RRACH motif distribution in exons 12 and 19 of CD44. (E) RIP assay showing m6A enrichment in exons 12 and 19 in NNMT‐KD QGY‐7701 and NNMT‐overexpressing MHCC‐97H cells, respectively. (F–H) Co‐IP assay showing protein–protein binding. (I) Representative IHC images showing subcellular location and intensity of NNMT and of CD44v3 in NNMT‐high and NNMT‐low patient samples. (J). Regression analysis of CD44v3 and NNMT protein expression based on IHC IDO in HCC tissues. The *t*‐test was used in E, G, H. The error bars represent SD. Scale bars = 200 μm (black) or 40 μm (white). Each experiment was performed in triplicate. ***P < 0.001, **P < 0.01, and *P < 0.05.

Various CD44 splice variants are associated with tumor progression in epithelial‐type carcinomas including liver cancer (Dhar *et al.*, 2018). Specific primers were designed to detect the expression of different CD44 variants in SMMC‐7721 cells and showed relative abundance of the V3/V4/V6 isoforms (Fig. 4A). In addition, the CD44v3 isoform was upregulated in QGY‐7701 cells cocultured with activated HSCs (Fig. 4B). Consistent with this, NNMT overexpression in MHCC‐97H and SMMC‐7721 cells also upregulated CD44v3, without affecting CD44v4 levels (Fig. 4C). Several factors involved in regulating alternative splicing are closely related to m6A modification (Pendleton *et al.*, 2017). Taking both into consideration, we hypothesized that NNMT regulated CD44 splicing by altering the CD44 mRNA m6A pattern, which increased the expression of CD44v3. Sequence analysis revealed multiple RRACH motifs in exon 12 adjacent to the CD44v3 splice site and in exon 19 adjacent to the stop codon (Fig. 4D).

To verify whether NNMT altered the abundance of m6A in both exons, we performed the RIP assay using monoclonal antibody against m6A and a rabbit IgG antibody as the control. Interestingly, the m6A abundance in both exons showed a significant negative correlation with NNMT expression levels (Fig. 4E). Furthermore, co‐IP assay showed that NNMT minimally affected the expression levels of the m6A demethylase ALKBH5 and FTO (Fig. 4F–G), but augmented their binding to m6A (Fig. 4H). In addition, no obvious binding of NNMT to m6A was observed (Fig. 4F), but a significant positive correlation was seen between CD44v3 and NNMT protein levels in HCC tissues (Fig. 4I–J). Taken together, NNMT impaired mRNA m6A modification indirectly by diminishing the intracellular methylation potential (SAM/SAH), thereby facilitating CD44v3 formation.

### 3.7 The NNMT product 1‐MNA prevents ubiquitin‐mediated CD44 degradation

**Figure 5:**
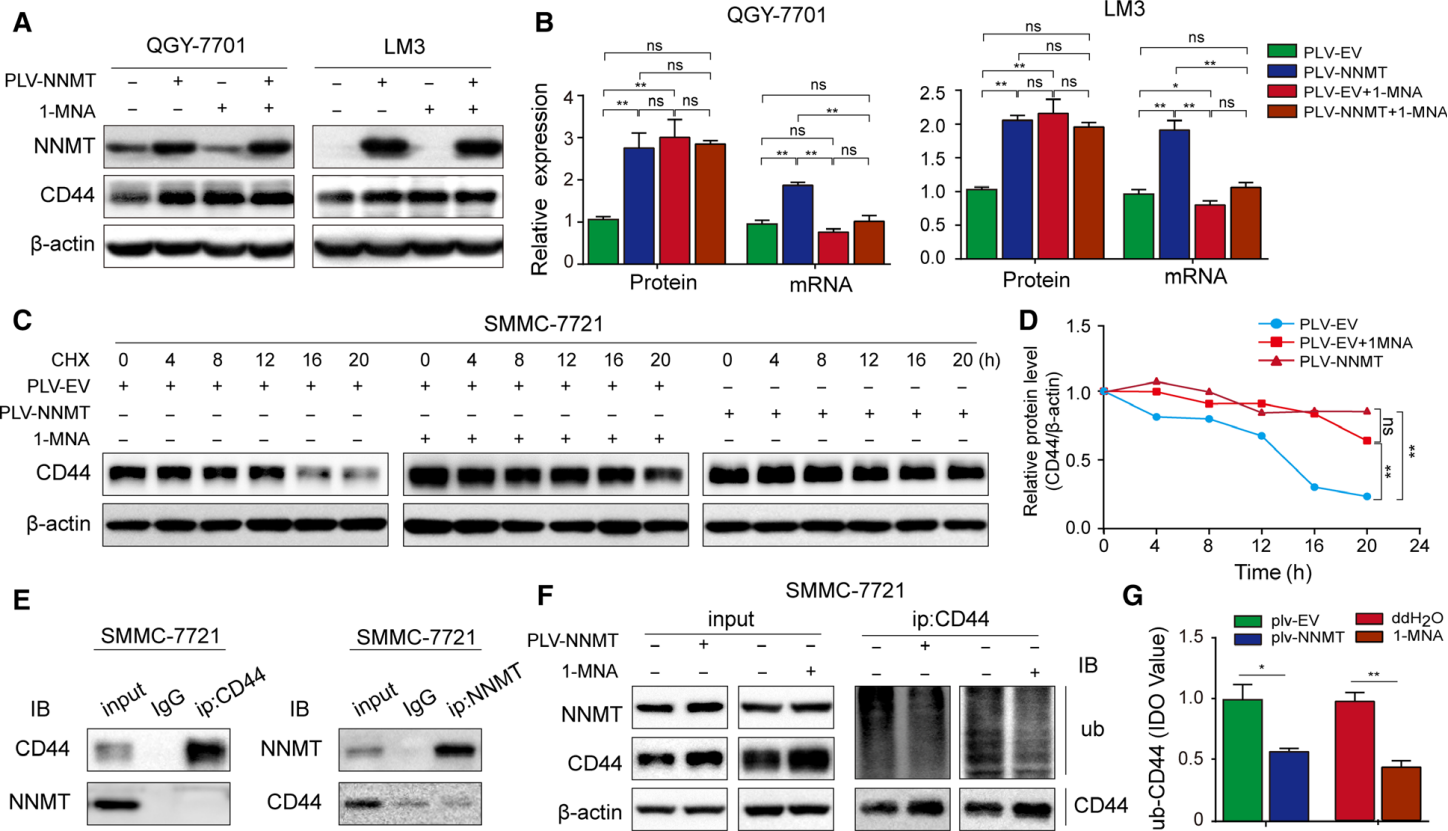
1‐MNA prevents ubiquitin‐mediated CD44 degradation. (A‐B) Effect of 1‐MNA on CD44 protein and mRNA levels in QGY‐7701 and LM3 cells. (C) CD44 protein levels in control, NNMT‐overexpressing and 1‐MNA‐treated SMMC‐7721 cells after treatment with CHX (20 µg·mL^−1^ for 20 h). (D) CD44 protein degradation curve in the above three groups. (E) Co‐IP assay showing NNMT‐CD44 binding. (F‐G) Co‐IP and immunoblots showing CD44 ubiquitination levels in control, NNMT‐overexpressing and 1‐MNA‐treated SMMC‐7721 cells. The *t*‐test was used in B, G. The error bars represent SD. Each experiment was performed in triplicate. ***P < 0.001, **P < 0.01, and *P < 0.05.

The catalytic product of NNMT is 1‐MNA, which suppresses NNMT activity via a negative feedback mechanism (Hwang and Song, 2017). Therefore, we hypothesized that 1‐MNA inhibited the NNMT‐induced CD44 upregulation. Surprisingly, however, while 1‐MNA decreased CD44 mRNA levels in QGY‐7701 and LM3 cells, it did not significantly affect its protein levels (Fig. 5A–B). This strongly indicates that 1‐MNA regulates the stability of CD44 protein. Therefore, we measured the rate of CD44 degradation in SMMC‐7721 cells treated with cycloheximide (CHX), an inhibitor of protein translation, and found that the CD44 degradation rate was slower in both NNMT‐overexpressing and 1‐MNA‐treated cells compared to the controls (Fig. 5C–D). Although there was no remarkable association between NNMT and CD44 protein (Fig. 5E), both NNMT‐overexpressing and 1‐MNA‐treated SMMC‐7721 cells showed diminished CD44 polyubiquitination (Fig. 5F–G), indicating that NNMT can prevent ubiquitin‐mediated CD44 degradation via 1‐MNA.

### 3.8 Statins inhibit migration and invasiveness of hepatoma cells by targeting NNMT

**Figure 6:**
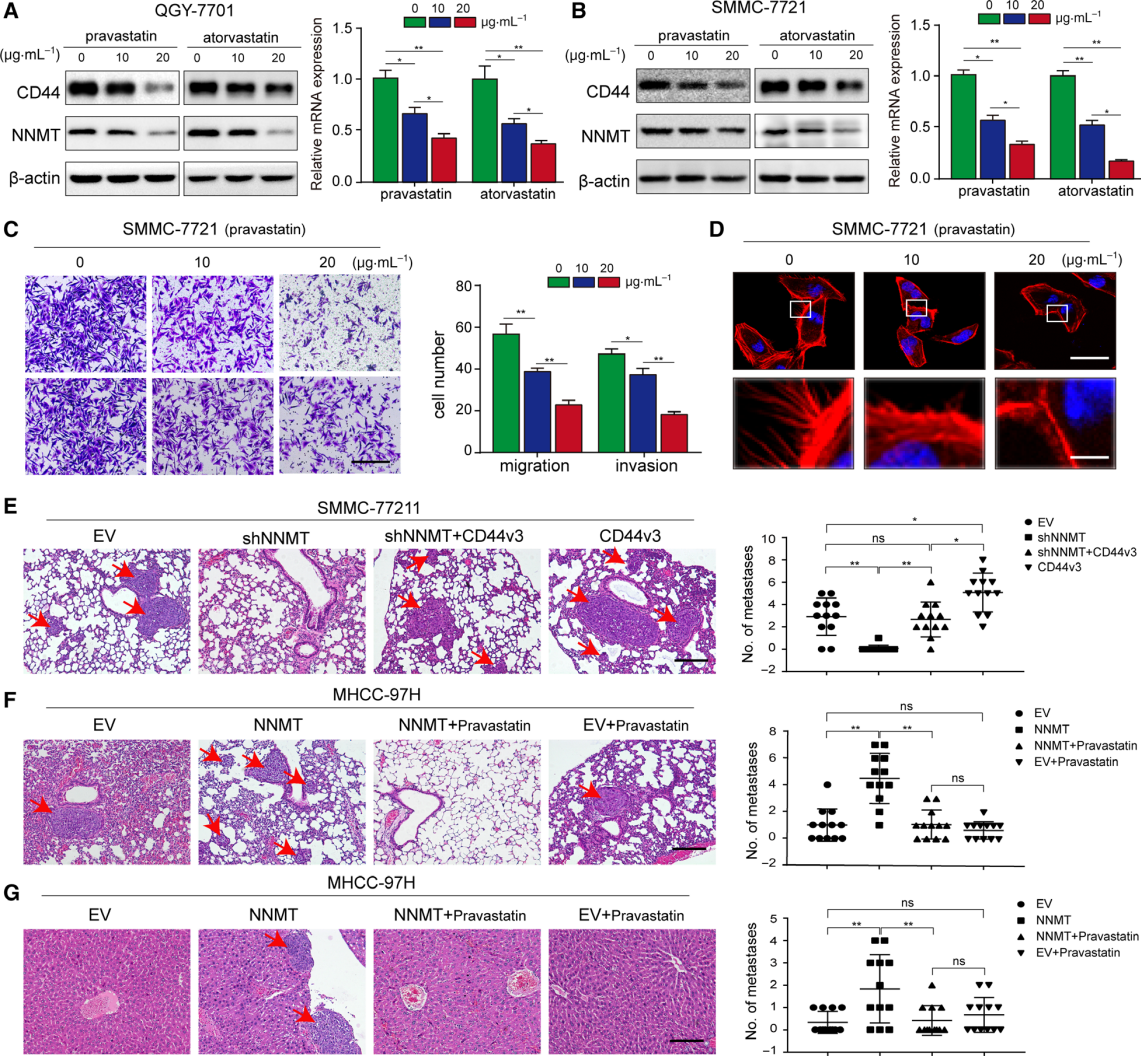
NNMT enhances tumor metastasis by regulating CD44v3 and is inhibited by pravastatin. (A–B) The effect of different doses of pravastatin and atorvastatin on NNMT protein and mRNA levels in SMMC‐7721 and QGY‐7701 cells. (C) Transwell assay showing the invasion and metastatic ability of SMMC‐7721 cells treated with different doses of pravastatin. (D) Representative CLSM images of phalloidin‐stained pravastatin‐treated SMMC‐7721 cells showing cytoskeletal rearrangement. Each experiment was performed in triplicate. (E). Effects of NNMT and CD44 overexpression on HCC lung metastasis in an orthotopic xenograft model (*n* = 12 per group). Metastatic tumors were analyzed by HE staining. (F–G) Effects of statins on NNMT‐induced lung and liver metastasis in an atopic xenograft model (*n* = 12 per group). Nodules with diameter > 2 mm were identified as metastatic tumors and counted. The *t*‐test was used in A, B, C, and the error bars represent SD. The nonparametric test was used in E, F, G, and the error bars represent SD. Scale bars = 200 μm.***P < 0.001, **P < 0.01, and *P < 0.05.

Based on the present and previous findings (Kim *et al.*, 2009; Mu *et al.*, 2013), we surmised that NNMT promotes invasion and metastasis in liver cancer. Given that NNMT also affects lipid metabolism which is closely related to tumor survival (Hong *et al.*, 2015), we examined the effects of common lipid‐lowering drugs on NNMT expression and found a significant inhibitory effect of statins. As shown in Fig. 6A–B, both pravastatin and atorvastatin significantly downregulated NNMT protein and mRNA levels in a dose‐dependent manner in QGY‐7701 and SMMC‐7721 cells. Likewise, CD44 expression levels, migration, and invasiveness but not cell activity of liver cancer cells was affected (Figs 6C and S6A). In addition, phalloidin staining of the cytoskeleton showed a gradual decline in microfilament density with increasing drug dose (Fig. 6D), indicating that statins not only inhibit NNMT expression but also the motility of hepatoma cells.

### 3.9 NNMT enhances tumor metastasis via CD44v3 and is inhibited by pravastatin *in vivo*

We analyzed the effect of NNMT on HCC cell metastasis in an orthotopic xenograft model, which was established by transplanting SMMC‐7721 cells transfected with plko‐EV (empty vector), plko‐shNNMT, plko‐shNNMT + plv‐CD44v3, and plv‐CD44v3 in the liver subcapsule of nude mice. Forty‐five days after implantation, the mice were euthanized and the metastatic nodules in the lungs and liver were counted. NNMT KD significantly inhibited the metastatic ability of the HCC cells, while overexpressing CD44v3 reversed this inhibitory effect. Conversely, CD44v3 overexpression alone heightened the metastatic ability of SMMC‐7721 cells (Fig. 6E). Taken together, these findings indicate that NNMT enhanced *in vivo* tumor metastasis via CD44v3.

To determine the *in vivo* effect of statins on NNMT‐induced metastasis, we established an atopic xenograft model of HCC by subcutaneously transplanting nude mice with equal numbers of MHCC‐97H cells transfected with either EV or NNMT, and injected them with either pravastatin or 0.9% saline intravenously every 7 days (four groups in total: EV + saline, NNMT + saline, EV + pravastatin and NNMT + pravastatin). Two months later, the mice were sacrificed and the metastatic nodules in the lungs and liver were counted (Fig. 6F–G). The mice injected with NNMT‐overexpressing cells developed more pulmonary and intrahepatic micrometastatic nodules and showed a higher metastasis ratio compared to mice injected with control cells. Pravastatin injection attenuated the NNMT‐induced metastasis, but failed to retard metastatic growth independent of NNMT expression in mice injected with MHCC‐97H‐EV cells. Therefore, we hypothesized that pravastatin inhibited the metastatic ability of HCC cells by targeting NNMT. In addition, the OS of the different groups corresponded to their respective metastatic loads (Fig. S7A–B). In conclusion, NNMT enhanced tumor metastasis by regulating CD44v3, and pravastatin inhibited this effect.

## 4 Discussion

Although the role of the tumor microenvironment in tumor progression is well established (Amicone and Marchetti, 2018; Kamil and Rowe, 2018), the cross‐talk between HSC and HCC cells in liver cancer metastasis has hardly been explored. Our previous study showed that activated HSCs create an immunosuppressive microenvironment in an orthotopic liver tumor mouse model by inducing expansion of the Tregs and MDSCs (Zhao *et al.*, 2011) (Zhao *et al.*, 2014). However, whether and how activated HSCs promote liver cancer metastasis via epigenetic modifications remains unclear.

In the present study, we found that activated HSCs upregulated NNMT expression levels in hepatoma cells, based on our previous study and other reports, which is most likely the result of TGF‐β secretion by the HSCs (Campagna *et al.*, 2018; Coulouarn and Clement, 2014; Liang *et al.*, 2018). As shown in Fig. S8, TGF‐beta can indeed upregulate the expression of NNMT in hepatoma cells, and the mechanism will continue to be further studied. NNMT methylates NAM and is expressed at high levels in several cancers (Liang *et al.*, 2018; Pozzi *et al.*, 2018; Song *et al.*, 2017; You *et al.*, 2018) like renal clear cell carcinoma, bladder cancer, and the gastric and colorectal cancers. NNMT KD has similarly been associated with the inhibition of proliferation and/or metastasis of oral squamous carcinoma, renal clear cell carcinoma cells, Panc‐1 pancreatic cancer cells, etc. Intriguingly, we detected lower NNMT levels in HCC tissues relative to normal adjacent tissues in agreement with previous report (Aksoy *et al.*, 1994). However, NNMT was still abundant in liver cancer tissues and was positively correlated with tumor vascular invasion, high serum HBV levels, and distant metastasis, and was a predictor for OS and recurrence‐free survival in HCC patients. In addition, NNMT increased HCC cell invasion and tumor metastasis by enhancing CD44v3 expression.

Although the functional role of NNMT in various cancers has been ascertained, the exact mechanism of its action is unknown. Ulanovskaya et al proposed that NNMT acts as a methyl donor sink (Ulanovskaya *et al.*, 2013) and alters the intracellular methylation potential (SAM/SAH ratio) and therefore the epigenetic state of the cells. Consistent with this, global H3 methylation on lysine 9 (H3K9) and lysine 27 (H3K27) was decreased in renal clear cell carcinoma cells overexpressing NNMT (Ulanovskaya *et al.*, 2013). In contrast, KD of NNMT in ovarian carcinoma cells had the opposite effect as it increased the SAM/SAH ratio, as well as global H3K9 and H3K27 trimethylation. Similar changes in methylation state were also observed in one nonhistone protein and protein phosphatase 2, indicating cellular targets other than histones that might be differentially methylated by changes in NNMT expression and methyl donor levels (Palanichamy *et al.*, 2017). Given that NNMT has no effect on the SAM/SAH ratio in hepatocytes due to the high intrinsic level of GNMT (Liao *et al.*, 2012), we first determined whether NNMT affected the methylation potential of liver cancer cells. NNMT overexpression lowered the intracellular SAM/SAH ratio while its KD had the opposite effect, indicating that unlike in normal liver cells, NNMT regulated the epigenetic landscape of HCC cells. This can be explained by the lower levels/absence of GNMT in HCC cells and tissues relative to normal cells. In addition, NNMT and GNMT did not regulate each other’s expression levels. Thus, GNMT competitively inhibits NNMT in terms of methylation donor regulation in normal liver cells but not in HCC cells.

The cell adhesion molecule CD44, especially its V3 and V6 variants, is abundantly expressed on tumor cells and confers an invasive phenotype. CD44 expression level is therefore correlated with tumor grade, lymph node invasion, metastatic spread, and overall prognosis (Sagawa *et al.*, 2016). In addition, CD44 is also a marker of stem cells that can be positively regulated by NNMT (Jung *et al.*, 2017), although the mechanistic basis is unknown. Therefore, we further explored whether NNMT regulated CD44 expression via methylation. NNMT overexpression in HCC cells decreased H3K27 trimethylation in addition to lowering the SAM/SAH ratio, which increased CD44 transcription. At the same time, CD44v3 formation was induced by the decrease in CD44 m6A methylation due to increased demethylase (FTO and ALKBH5) interaction with m6A, although the total expression levels of the demethylases did not change significantly. To better understand the role of NNMT in promoting liver cancer metastasis, it is necessary to determine which RNA methylases are involved in this process and how the demethylases are recruited.

The enzymatic activity of NNMT is inhibited by 1‐MNA, its catalytic product, via a feedback mechanism. However, we found that while it only partially inhibited NNMT activity, 1‐MNA significantly stabilized the CD44 protein by preventing ubiquitin‐mediated degradation. Shangyu Hong et al have reported that NNMT and its catalytic products are closely related to lipid metabolism (Hong *et al.*, 2015). Therefore, we explored the effect of common cholesterol‐lowering drugs on NNMT expression and found that NNMT was a target of statins that inhibited metastasis of hepatoma cells *in vitro* and *in vivo*. The exact underlying mechanism, vis‐a‐vis direct or indirect reaction to regulate metabolic changes, is the topic of our future study.

**Figure 7:**
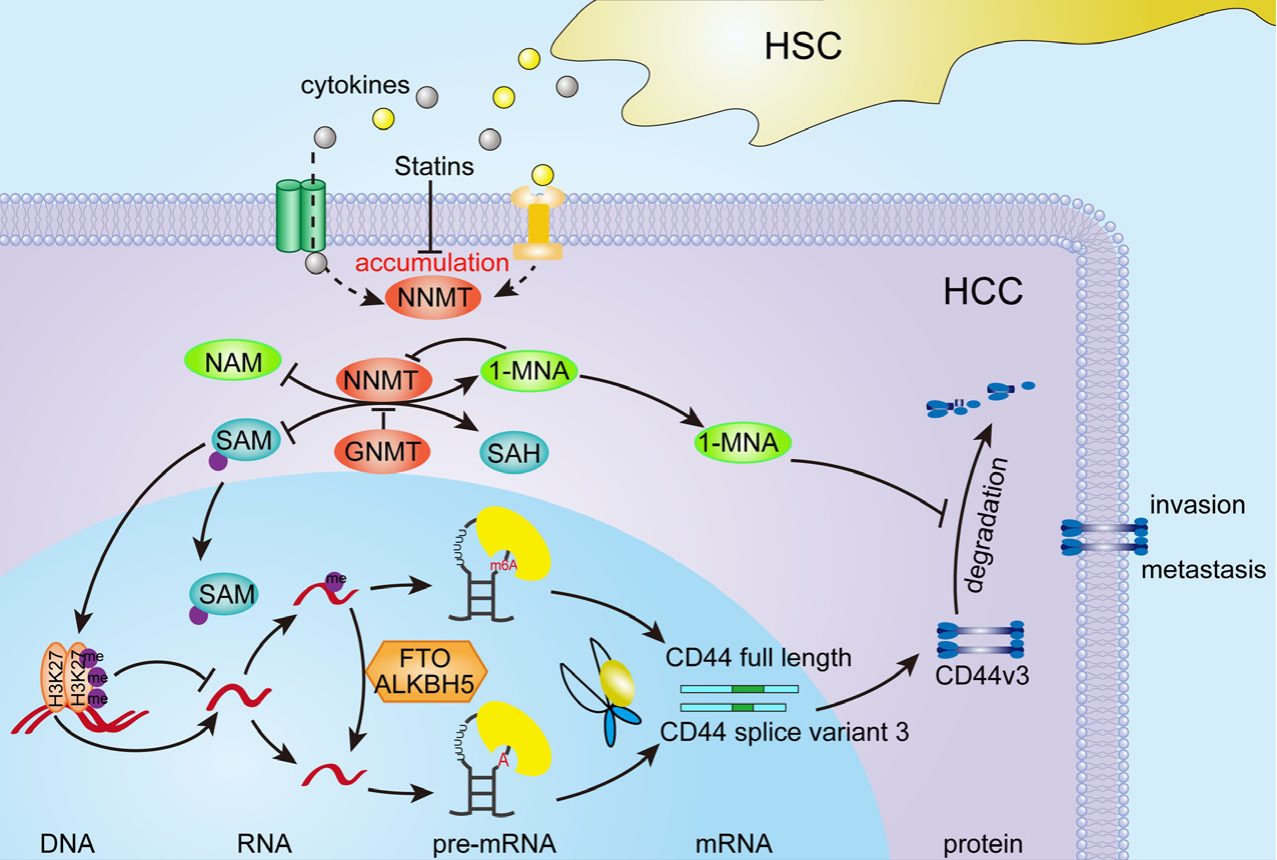
Schematic model of the stimulatory role of NNMT in HCC metastasis. Activated HSCs upregulated NNMT expression levels in hepatoma cells, which is most likely the result of cytokines (e.g., TGF‐β) secreted by the HSCs. High levels of NNMT altered intracellular methylation potential and decreased histone H3K27 methylation, which upregulated CD44 transcription. In addition, NNMT‐mediated CD44 mRNA m6A modification induced the CD44v3 splice variant, while its product 1‐MNA stabilized the CD44 protein by preventing ubiquitin‐mediated degradation. Finally, NNMT was a target of the antimetastatic statins.

To summarize, we have shown that activated HSCs induced aberrantly high levels of NNMT in HCC cells, which was correlated with an invasive phenotype. In addition, NNMT promoted migration, invasion, and metastasis of HCC cells *in vitro* and *in vivo*. Mechanistically (Fig. 7), the altered intracellular methylation potential decreased histone H3K27 methylation, which upregulated CD44 transcription. NNMT‐mediated CD44 mRNA m6A modification induced the CD44v3 splice variant, while its product 1‐MNA stabilized the CD44 protein by preventing ubiquitin‐mediated degradation. Finally, NNMT was a target of the antimetastatic statins. Taken together, our study provides evidence for the first time on the potential role of the NNMT/CD44v3 axis in regulating HCC metastasis and presents NNMT as a potential prognostic marker and therapeutic target for HCC. But there are still many limitations. First, no more conclusive evidence was obtained in transgenic mice due to conditions. Secondly, how to regulate the expression of NNMT in HCC and the factors affecting the NNMT/CD44 axis also requires further in‐depth research. At the same time, we also expect more interested researchers to join this study.

## 5 Conclusions

Taken together, our study provides evidence for the first time on the potential role of the NNMT/CD44v3 axis in regulating HCC metastasis and presents NNMT as a promising prognostic biomarker and therapeutic target for HCC.

## Conflict of interest

The authors declare no conflict of interest.

## Author contributions

LJ, YS, and ZS designed the experiments, analyzed and interpreted the data, and drafted and wrote the manuscript. HQ, WF, and XC performed plasmid cloning, cell line generation, and related experiments. CX and ZW collected clinical samples and also performed some experiments. ZC and YY performed data preprocessing. LJ and ZX analyzed the data and also performed some experiments. ZY, LP, ZY, WX, and LQ collected the data and performed some experiments. YZ and WX participated in the study designing and manuscript writing and were responsible for coordinating the study.

## Supporting information


**Fig S1.** NNMT expression in matched HCC and corresponding adjacent tissues. (A–B) *In situ* NNMT expression in 93 HCC and corresponding adjacent tissues (original magnification ×200), and the IOD values based on which 92 patients (excluding one case of loss during follow‐up) were divided into the NNMT‐high (*n *= 46) and NNMT‐low (*n *= 46) groups. (C) Immunoblots showing NNMT protein expression in paired HCC tumor and adjacent tissues. Scale bars = 200 μm (black) or 40 μm (white).

**Fig S2.** Heat map and Regression analysis. (A) Heat map showing the 19 common genes changed in SMMC‐7721 cells incubated with HSC‐CM and NNMT‐KD PLC/PRF/5 cells. (B) Regression analysis of CD44 and NNMT mRNA expression in hepatoma cell lines (LO2 and ATCC‐HepG2 cell lines included).

**Fig S3.** Mass spectrometric detection of relative SAM and SAH content and their ratio in NNMT‐KD LO2 and ATCC‐HepG2 cells. (A–B) Mass spectrometric detection of relative SAM and SAH content and their ratio in NNMT‐KD LO2 and ATCC‐HepG2 cells. The *t*‐test was used in A, B, C and the error bars represent SD.

**Fig S4.** Luciferase activity and the stability assay of CD44 mRNA. (A) CD44 promoter‐driven luciferase activity in 293T cells transfected with NNMT KD or overexpressing plasmids. (B) CD44 mRNA levels in NNMT‐KD or overexpressing cells after treatment with actinomycin D (2 µm). The *t*‐test was used in A, B, C and the error bars represent SD.

**Fig S5.** ChIP‐qPCR analyses of NNMT binding on CD44 promoter. (A‐B) ChIP‐qPCR analyses of NNMT binding on CD44 promoter in NNMT‐MHCC‐97H and NNMT‐KD SMMC‐7721 cells. The *t*‐test was used in A, B, C and the error bars represent SD.

**Fig S6.** The viability of SMMC‐7721 cells treated with pravastatin and atorvastatin. (A) CCK‐8 assay showing the viability of SMMC‐7721 cells treated with different concentrations of pravastatin and atorvastatin. The nonparametric test was used in E, F, G and the error bars represent SD.

**Fig S7.** The OS of the different groups. (A‐B). The OS of the different groups corresponded to their respective metastatic loads.

**Fig S8.** TGF‐beta upregulates the expression of NNMT. Immunoblot showing TGF‐beta can upregulate the protein expression of NNMT.

**Table S1.** List of primers sequences.
