# Supplementary figures and images for: Elevated N‐methyltransferase expression induced by hepatic stellate cells contributes to the metastasis of hepatocellular carcinoma via regulation of the CD44v3 isoform
Source: Mol Oncol. 2019 Jul 11;13(9):1993–2009. doi: 10.1002/1878-0261.12544 (PMC6717763; doi:10.1002/1878-0261.12544)

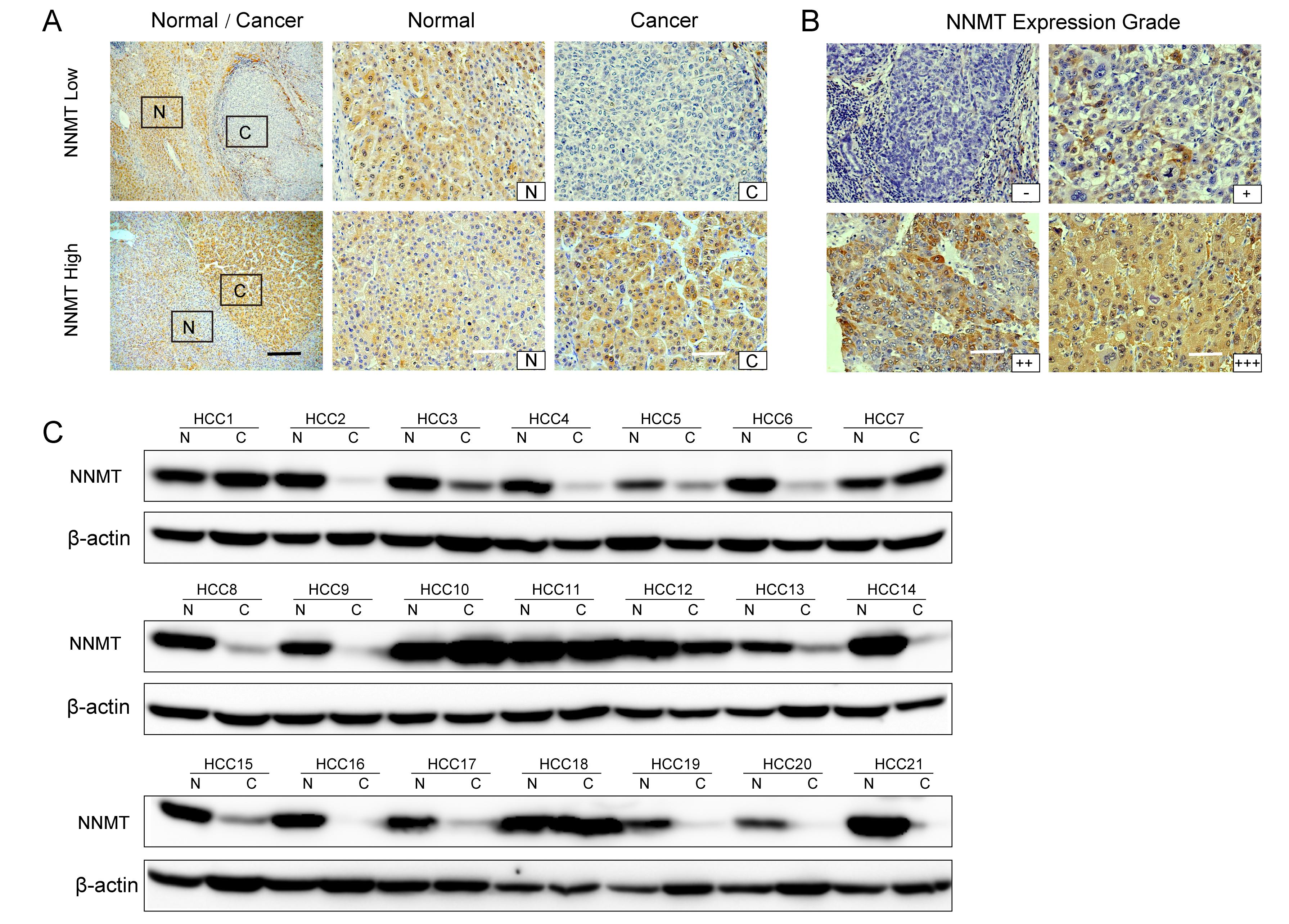

Supplement: Supplementary file 1 — Fig S1. NNMT expression in matched HCC and corresponding adjacent tissues. (A–B) In situ NNMT expression in 93 HCC and corresponding adjacent tissues (original magnification ×200), and the IOD values based on which 92 patients (excluding one case of loss during follow‐up) were divided into the NNMT‐high (n = 46) and NNMT‐low (n = 46) groups. (C) Immunoblots showing NNMT protein expression in paired HCC tumor and adjacent tissues. Scale bars = 200 μm (black) or 40 μm (white). [file MOL2-13-1993-s001.tif]

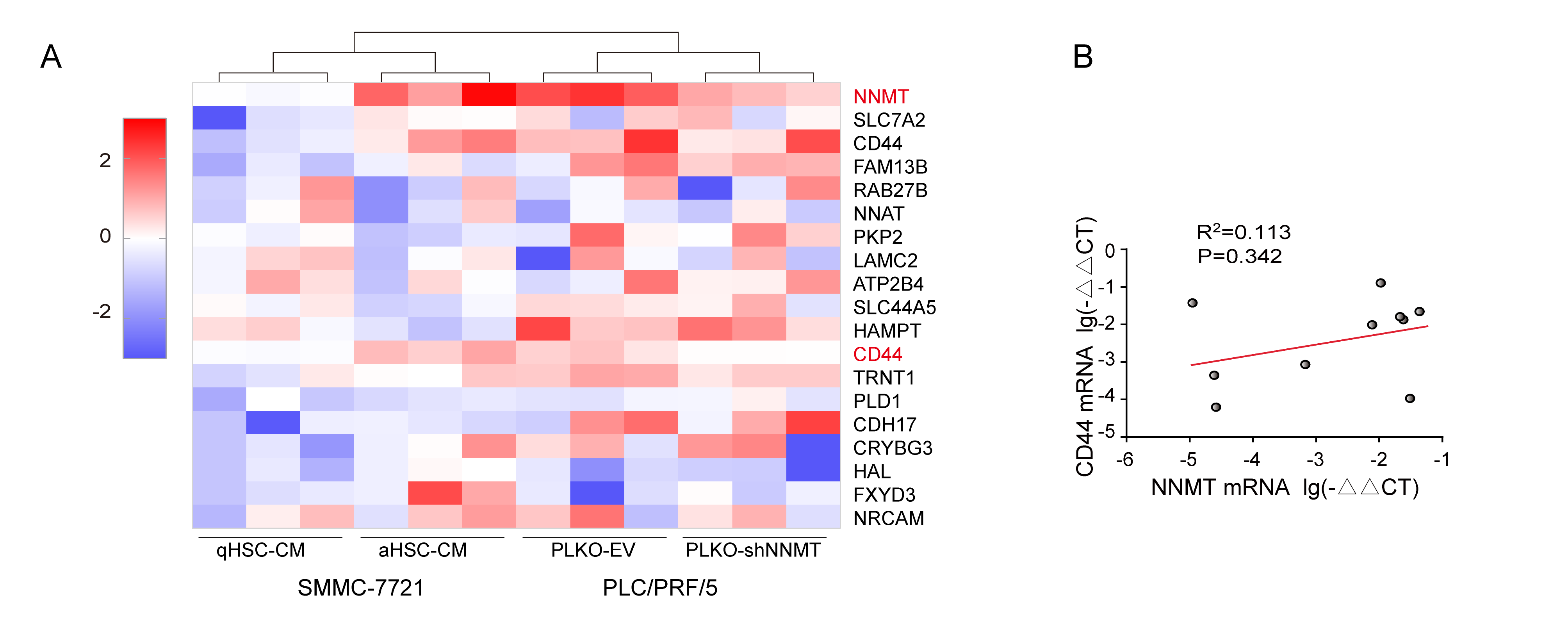

Supplement: Supplementary file 2 — Fig S2. Heat map and Regression analysis. (A) Heat map showing the 19 common genes changed in SMMC‐7721 cells incubated with HSC‐CM and NNMT‐KD PLC/PRF/5 cells. (B) Regression analysis of CD44 and NNMT mRNA expression in hepatoma cell lines (LO2 and ATCC‐HepG2 cell lines included). [file MOL2-13-1993-s002.tif]

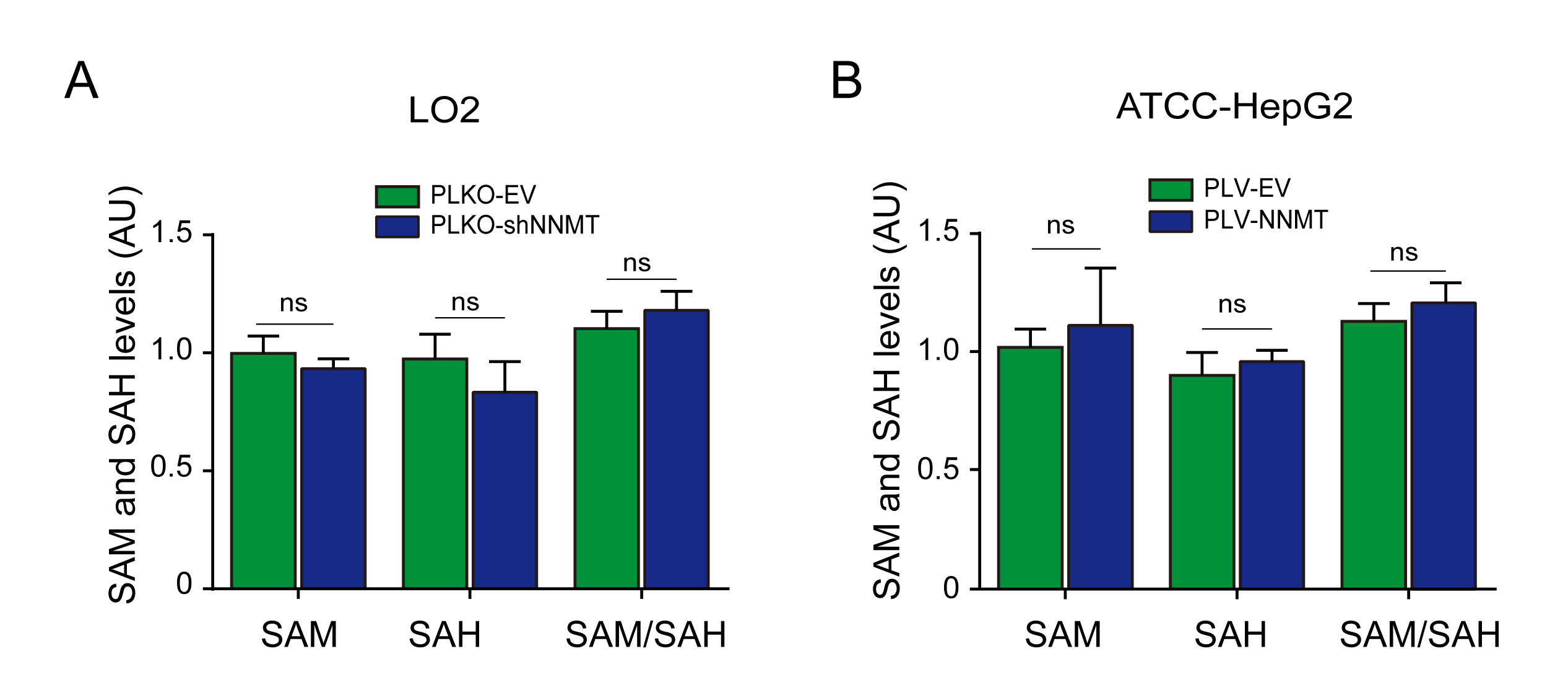

Supplement: Supplementary file 3 — Fig S3. Mass spectrometric detection of relative SAM and SAH content and their ratio in NNMT‐KD LO2 and ATCC‐HepG2 cells. (A–B) Mass spectrometric detection of relative SAM and SAH content and their ratio in NNMT‐KD LO2 and ATCC‐HepG2 cells. The t‐test was used in A, B, C and the error bars represent SD. [file MOL2-13-1993-s003.tif]

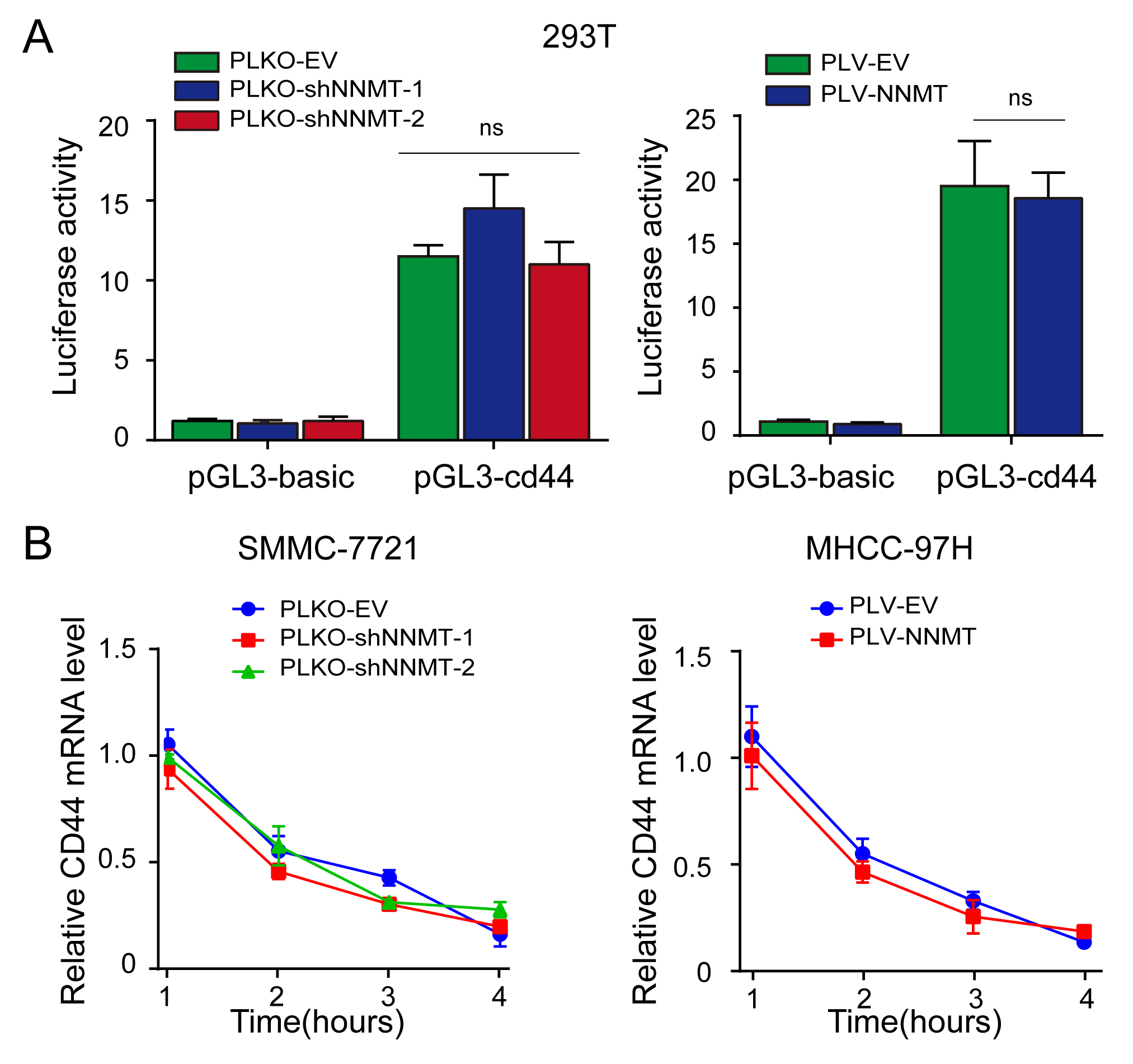

Supplement: Supplementary file 4 — Fig S4. Luciferase activity and the stability assay of CD44 mRNA. (A) CD44 promoter‐driven luciferase activity in 293T cells transfected with NNMT KD or overexpressing plasmids. (B) CD44 mRNA levels in NNMT‐KD or overexpressing cells after treatment with actinomycin D (2 µm). The t‐test was used in A, B, C and the error bars represent SD. [file MOL2-13-1993-s004.tif]

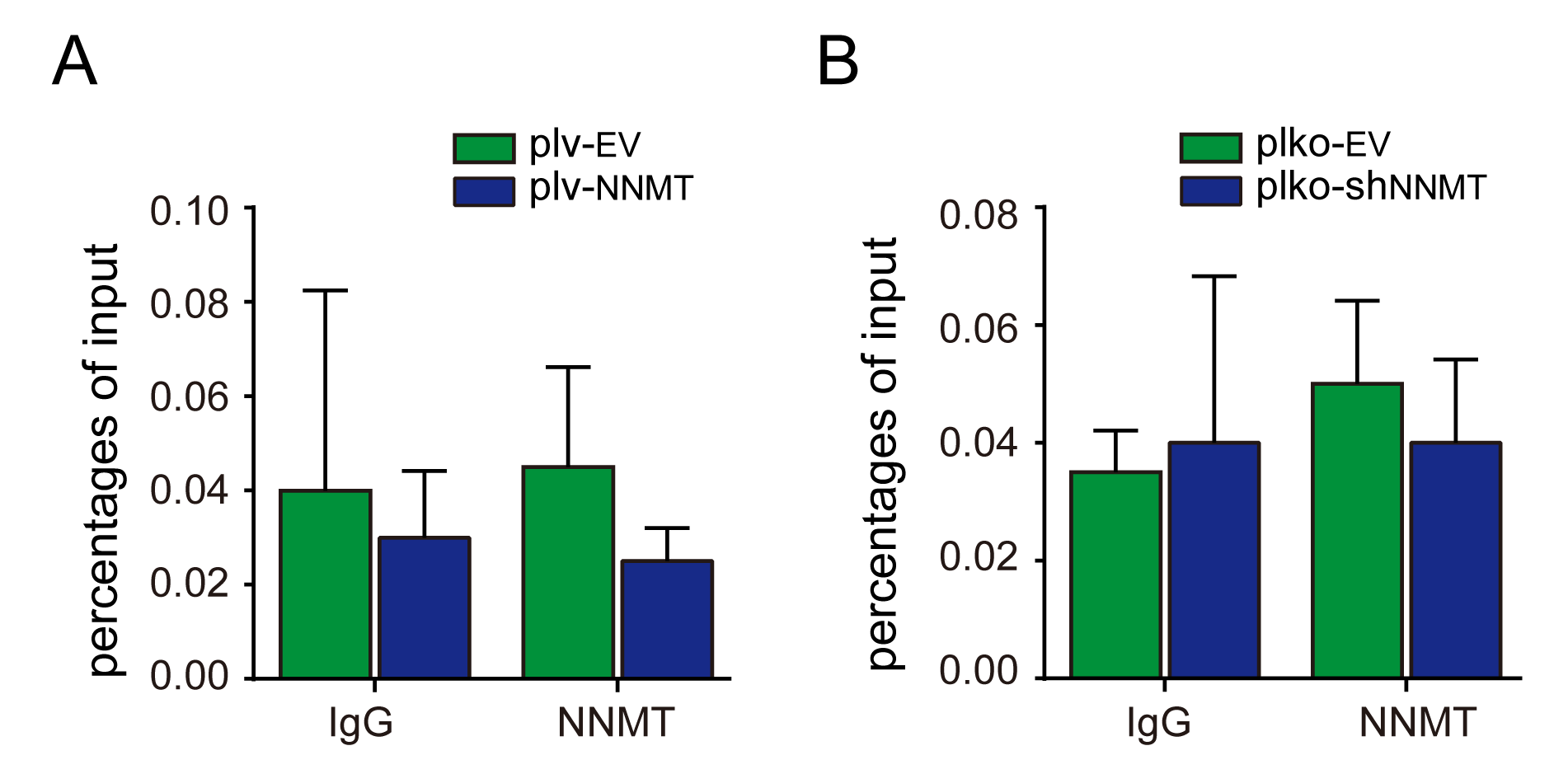

Supplement: Supplementary file 5 — Fig S5. ChIP‐qPCR analyses of NNMT binding on CD44 promoter. (A‐B) ChIP‐qPCR analyses of NNMT binding on CD44 promoter in NNMT‐MHCC‐97H and NNMT‐KD SMMC‐7721 cells. The t‐test was used in A, B, C and the error bars represent SD. [file MOL2-13-1993-s005.tif]

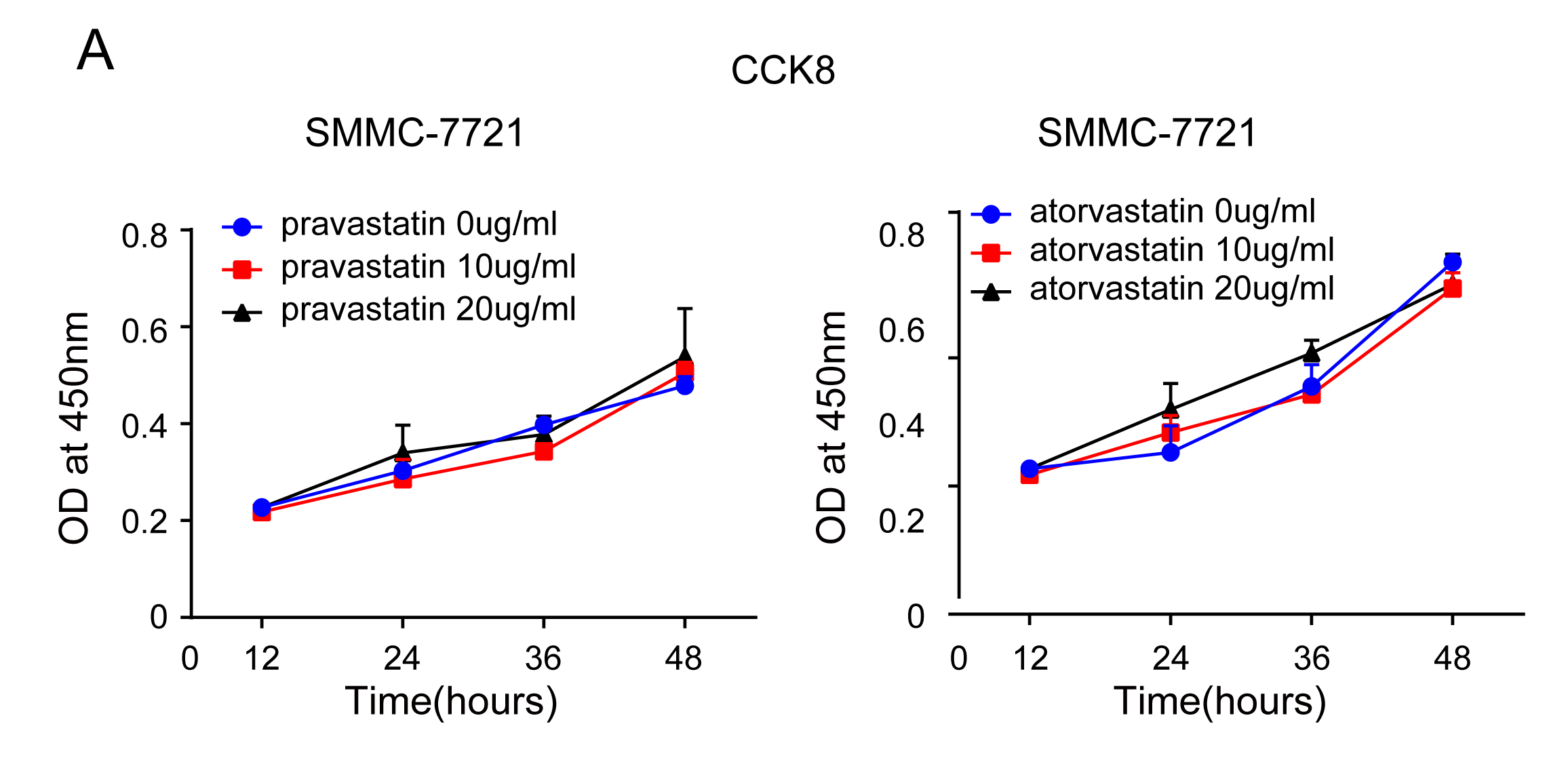

Supplement: Supplementary file 6 — Fig S6. The viability of SMMC‐7721 cells treated with pravastatin and atorvastatin. (A) CCK‐8 assay showing the viability of SMMC‐7721 cells treated with different concentrations of pravastatin and atorvastatin. The nonparametric test was used in E, F, G and the error bars represent SD. [file MOL2-13-1993-s006.tif]

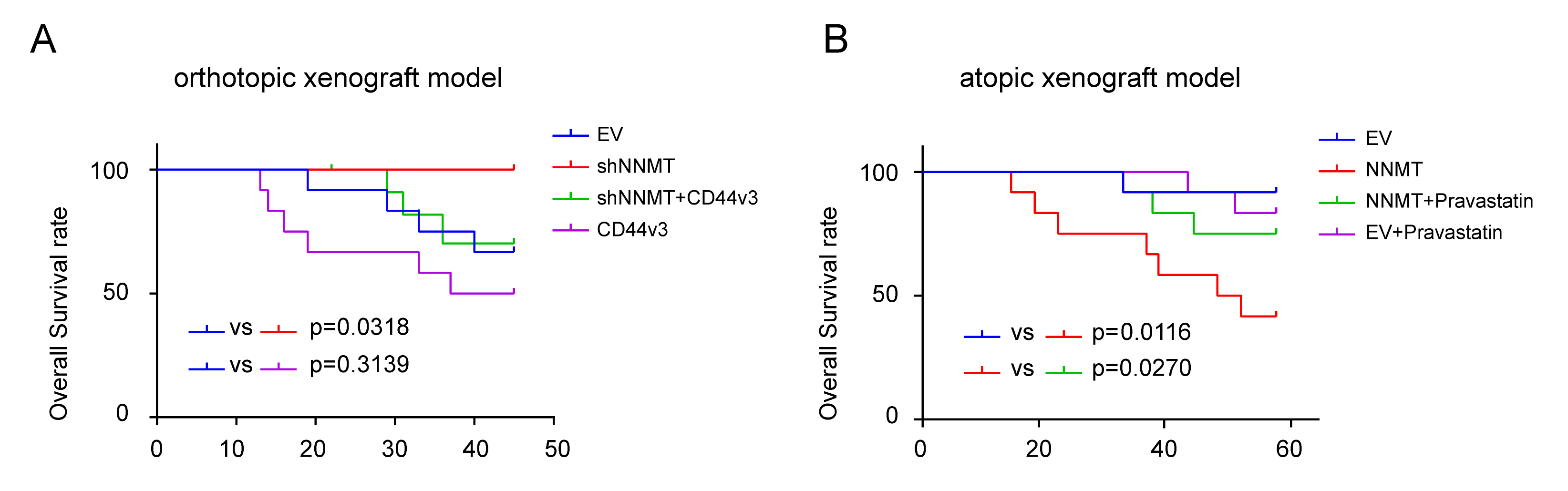

Supplement: Supplementary file 7 — Fig S7. The OS of the different groups. (A‐B). The OS of the different groups corresponded to their respective metastatic loads. [file MOL2-13-1993-s007.tif]

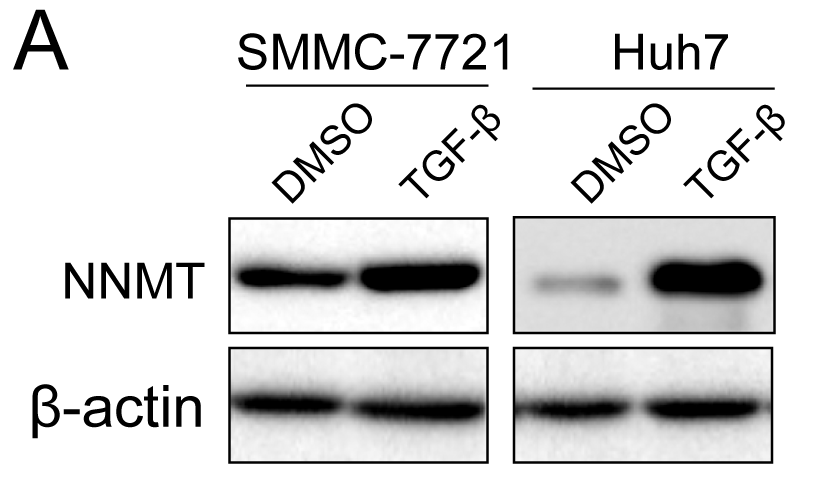

Supplement: Supplementary file 8 — Fig S8. TGF‐beta upregulates the expression of NNMT. Immunoblot showing TGF‐beta can upregulate the protein expression of NNMT. [file MOL2-13-1993-s008.tif]
