# Supplementary material for: Elevated N‐methyltransferase expression induced by hepatic stellate cells contributes to the metastasis of hepatocellular carcinoma via regulation of the CD44v3 isoform
Source: Mol Oncol. 2019 Jul 11;13(9):1993–2009. doi: 10.1002/1878-0261.12544 (PMC6717763; doi:10.1002/1878-0261.12544)
Supplement: Supplementary file 9 — Table S1. List of primers sequences. [file MOL2-13-1993-s009.docx]

|  | **Primer name** | **F:5’-3’** | | **R:5’-3’** |
| --- | --- | --- | --- | --- |
| RT-PCR | NNMT | GAGATCGTCGTCACTGACTACT | CACACACATAGGTCACCACTG | |
|  | GNMT | GACAAGTGGGTCATCGAAGAAG | TGGCAAGTGAGCGAAACTGT | |
|  | CD44 | CTGCCGCTTTGCAGGTGTA | CATTGTGGGCAAGGTGCTATT | |
|  | CD44-V1 | AACCCTACTGATGATGACG | AATGGTGCTGGAGATAAA | |
|  | CD44-V2 | AACCCTACTGATGATGACG | AATGGTGCTGGAGATAAA | |
|  | CD44-V3 | CCACCTGAAGAAGATTGT | TTACTCTGCTGCTGCGTTGTC | |
|  | CD44-V4 | CCACCTGAAGAAGATTGT | TTACTCTGCTGCTGCGTTGTC | |
|  | CD44-V5 | GTCAACAGTCGAAGAAGGTG | TTCCAAGATAATGGTCTAGGTC | |
|  | CD44-V6 | AACCCTACTGATGATGACG | GTGTTTGCTCCACCTTCT | |
|  | CD44-V7 | AACCCTACTGATGATGACG | GTGTTTGCTCCACCTTCT | |
|  | CD44-V8 | CTTGGCTTTGATTCTTGC | CTTGGTTCAGGTAGGGAG | |
|  | ACTA2 | CGATAGAACACGGCATCATC | CATCAGGCAGTTCGTAGCTC | |
|  | COL1A1 | CGGTGTGACTCGTGCAGC | ACAGCCGCTTCACCTACAGC | |
|  | NPC1 | GCAGTGCCTACCGAGTATTT | GACACACCGAGGTTGAAGATAG | |
| ChIP | P1 | AAGAAAGCCAGTGCGTCTC | GCTCTGCTGAGGCTGTAAAT | |
|  | P2 | GGGCGGATGGAAGGATATTTAG | CTGTCATAGGGCTGGCATTT | |
|  | P3 | GATCTGCTGGGTAGGAAAGATG | CATTCTCTCATCCACCCATACG | |
|  | P4 | GGCAAGGTCACACAACTAAGA | GGGACAAGTAAGTCATCCACAG | |
|  | P5 | TTCAGTCCCAAACACTCTTCC | ACCACTGTCTAGTCCGGTATAA | |
| PGL3.0 | CD44 | CGGTACCCCTTAGGCAAGGTCACACAAC | CCTCGAGGGTGTCCGGAGCGAACGGA | |
| PLV-puro | NNMT | GCTCTAGAATGGAATCAGGCTTCACCTC | CCGCTCGAGTCACAGGGGTCTGCTCAGCTT | |
|  | GNMT | CGAATTCATGGTGGACAGCGTGTACCG | CGGATCCTCAGTCTGTCCTCTTGAGCAC | |
|  | CD44V3 | GCTCTAGAATGGACAAGTTTTGGTGGCACG | CCGAATTCTTACACCCCAATCTTCATGTCCA | |
| PLKO.1 | shCD44 | GATCCACTCCATCTGTGCAGCAAATTCAAGAGATTTGCTGCACAGATGGAGTTTTTTTG | AATTCAAAAAAACTCCATCTGTGCAGCAAATCTCTTGAATTTGCTGCACAGATGGAGTG | |
|  | shNNMT-1 | CCGGTAGGCTGGCTACACAATCGAATGGTTCAAGAGACCATTCGATTGTGTAGCCAGCCTTTTTTTG | AATTCAAAAAAAGGCTGGCTACACAATCGAATGGTCTCTTGAACCATTCGATTGTGTAGCCAGCCTA | |
|  | shNNMT-2 | CCGGTGTGGTGACCTATGTGTGTGATCTTTCAAGAGAAGATCACACACATAGGTCACCACTTTTTTG | AATTCAAAAAAGTGGTGACCTATGTGTGTGATCTTCTCTTGAAAGATCACACACATAGGTCACCAA | |
|  | shNNMT-3 | CCGGTAGGCTGGCTACACAATCGAATGGTTCAAGAGACCATTCGATTGTGTAGCCAGCCTTTTTTTG | AATTCAAAAAAAGGCTGGCTACACAATCGAATGGTCTCTTGAACCATTCGATTGTGTAGCCAGCCTA | |

**Table S1. Primers sequences.**
